# Ea/Ees and oscillatory power fraction capture complementary but distinct components of ventriculoarterial coupling: a porcine study

**DOI:** 10.3389/fphys.2026.1904487

**Published:** 2026-08-25

**Authors:** Hans Martin Flade, Idar Kirkeby-Garstad, Oddveig Lyng, Tomas Dybos Tannvik, Petter Aadahl, Andreas Østvik

**Affiliations:** 1 Department of Circulation and Medical Imaging, Faculty of Medicine and Health Sciences, Norwegian University of Science and Technology (NTNU), Trondheim, Norway; 2 Department of Anesthesia and Intensive Care, St Olav’s Hospital, Trondheim University Hospital, Trondheim, Norway; 3 Unit of Comparative Medicine, Norwegian University of Science and Technology (NTNU), Trondheim, Norway; 4 Medical Image Analysis, Department of Health Research, SINTEF Digital, Trondheim, Norway; 5 Department of Cardiology and Cardiothoracic Surgery, St Olavs Hospital, Trondheim University Hospital, Trondheim, Norway

**Keywords:** arterial load, cardiac power, elastance, hemodynamic monitoring, oscillatory power fraction, ventriculo-arterial coupling (VAC)

## Abstract

**Introduction:**

Ventriculoarterial coupling (VAC), traditionally assessed using the invasive “gold standard” Ea/Ees ratio (arterial elastance to end-systolic elastance), is clinically important but requires approximations that are impractical at the bedside. Oscillatory power fraction (OPF), defined as the ratio of oscillatory to total hydraulic power and derived from synchronized aortic flow–pressure measurements, has been proposed as a pragmatic VAC surrogate. This study aimed to clarify the possible correlation between VAC and OPF in pharmacologically induced hemodynamic extremes.

**Methods:**

In 14 juvenile pigs challenged with vasoactive (phenylephrine, nitroprusside) and cardioactive drugs (dobutamine, esmolol) titrated toward protocolized targets (± 40% MAP, ± 50% d*P*/d*t*), we obtained a comprehensive dataset using intracardiac conductance catheters, intraaortic pressure probes, and ascending aortic flow probes. Linear mixed models with animal as a random effect compared baseline and drug-effect states. The coupling of the two responses was assessed from each animal’s baseline-to-effect change, corrected for measurement error estimated from the replicate recordings.

**Results:**

Ea/Ees changed by 10%–36% across the four interventions, whereas OPF changed by 1.5%–12%, and under phenylephrine and dobutamine, the change in OPF was negligible (|*d*| < 0.2, both *p* > 0.2) despite measurable changes in Ea/Ees. Esmolol was the only challenge under which both indices changed substantially in the same direction (Ea/Ees: + 35.4%, OPF: + 12.2%). Whether the magnitudes of the two responses were coupled could not be determined. Dobutamine markedly increased Ees, total power, and oscillatory power while preserving OPF despite a reduction in Ea/Ees, indicating improved VAC but stable pulsatile efficiency. Vasoactive drugs (phenylephrine, nitroprusside) substantially altered Ea/Ees without relevant OPF changes.

**Conclusion:**

Ea/Ees and OPF are not interchangeable. OPF responded far less strongly than Ea/Ees to the loading changes imposed here and did not resolve them under two of the four challenges. This is consistent with the two indices reflecting predominantly steady and pulsatile aspects of arterial load, although the present data do not demonstrate that separation directly. While OPF cannot replace Ea/Ees, power-derived indices offer practical adjuncts for characterizing ventriculoarterial interactions and the effects of selected drugs at the intensive care bedside.

## 1 Introduction

Hemodynamic status at the bedside is traditionally assessed by considering cardiac performance and vascular properties as separate entities, using parameters such as blood pressure, heart rate, and cardiac output to characterize the hemodynamic state (Cecconi et al., 2014).

However, the effectiveness of the cardiovascular system depends not only on the performance of its individual components but also on the interaction between the ventricle as a pump and the arterial system as its hydraulic load.

Ventriculoarterial coupling (VAC) in general describes this interaction and reflects how efficiently the mechanical energy generated by the ventricle is transferred to the arterial circulation. Cardiovascular efficiency describes the energetic cost required to provide a given cardiac output and blood pressure (Guinot et al., 2022).

On the one hand, VAC is commonly expressed as the ratio between arterial elastance (Ea) and end-systolic elastance (Ees). It is considered an important marker of disease severity and is a strong independent predictor of outcomes in heart failure, hypoperfusion (Trambaiolo et al., 2022; Ky et al., 2013; Zhou et al., 2022), and trauma patients (Chang et al., 1998). Improving and optimizing VAC, as described by Ea/Ees, has been shown to improve circulation in critically unwell patients (Guarracino et al., 2018) and may improve survival (Chang et al., 2002).

Both Ea and Ees can be approximated by different methods using blood pressure measurements and comprehensive echocardiographic assessments.

The most widely used method is the single-beat approach by Chen et al. (2001). Ees is estimated using echocardiographically determined stroke volume and EF, hemodynamic parameters (diastolic and systolic blood pressure, preejection time, and total ejection time), and an estimated normalized ventricular elastance computed using a complicated polynomial formula. Unfortunately, this polynomial formula is very sensitive to small errors in the measurement of time intervals. In addition, this method has not been validated in different pathological hemodynamic states in humans.

Cardiac MRI as well as 3D echocardiography can be used to determine cardiac volumes and thereby assist in determining VAC by Ea/Ees (Lang et al., 2015). CMR-derived VAC accurately reflects invasively derived VAC in patients with repaired Tetralogy of Fallot (Shimfessel et al., n.d) and is a strong predictor of ventricular efficiency in patients with repaired Tetralogy of Fallot and those with Fontan circulation (Banga et al., n.d).

Various methods using different noninvasive echocardiographic assessments exist, all with varying degrees of validation, reproducibility, and established reference values. It is important to emphasize, though, that all these approaches rely on simplifying assumptions and provide non-interchangeable approximations (Wo et al., 2019; Ikonomidis et al., 2019). In the end, all these indices are indirect surrogates and do not directly quantify energy transfer. Accordingly, the European Society of Cardiology (ESC) concludes that the optimal marker to assess VAC is not yet established (Ikonomidis et al., 2019).

**Figure 1:**
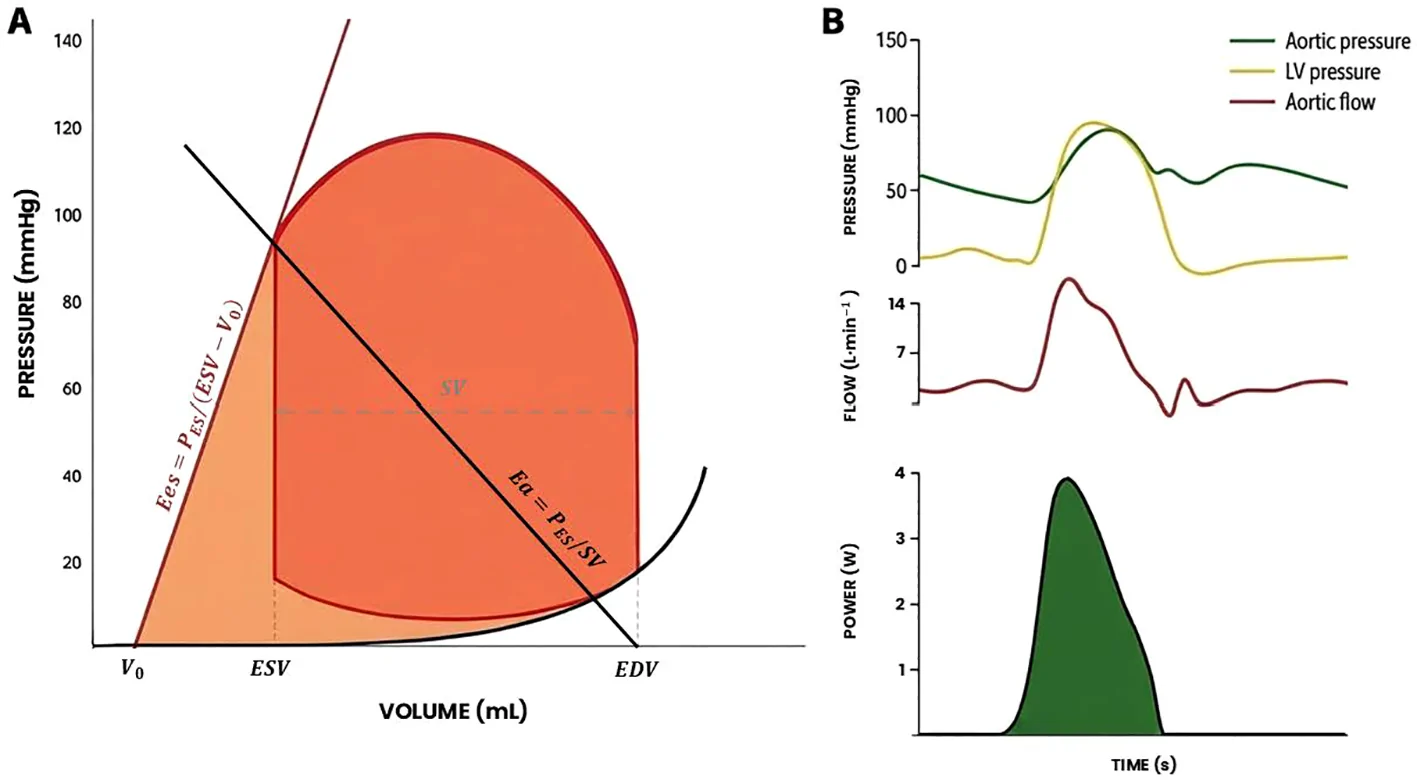
**(A)** Schematic PV loop. **(B)** Schematic visualization of the left ventricle pressure-flow integral yielding total power. LV, left ventricle; Ees, end-systolic elastance; Pes, end-systolic pressure; ESV, end-systolic volume; V0, volume in the depressurized ventricle; EDV, end-diastolic volume; SV, stroke volume; Ea, arterial elastance.

The energy provided during each ventricular contraction consists of *stroke work* (SW), representing energy delivered into the circulation, and *potential energy* (PE), referring to the energy dissipated during the cardiac cycle. Together, these form the pressure-volume area (PVA), which is linearly correlated with myocardial oxygen consumption (Takaoka et al., 1992). The ratio between SW and PVA represents left ventricular *energy efficiency* (Starling, 1993) (**Figure 1A**).

**Figure 2:**
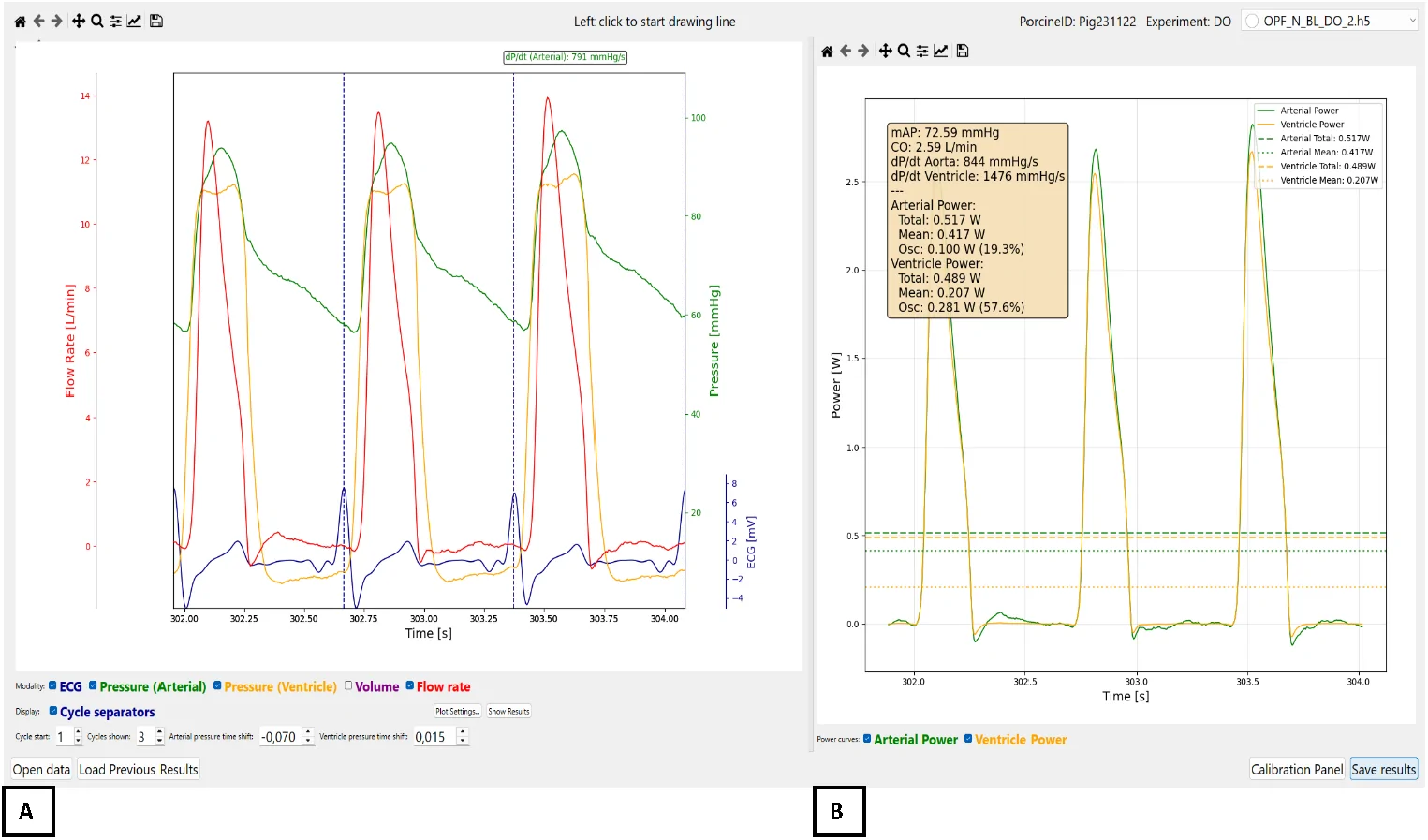
Raw aortic pressure and flow were analyzed using custom in-house software. Upstrokes of aortic pressure and transit-time aortic flow were manually aligned, and ventricular pressure was synchronized to diastolic pressure and aortic flow onset **(A)**. From the synchronized signals, total (tPWR), mean (mPWR), and oscillatory power (oPWR), d*P*/d*t*, and standard hemodynamic variables (arterial pressure, stroke volume, and cardiac output) were computed **(B)**.

In hydraulics, the product of pressure and flow constitutes power. Simplifying this, mean cardiac power output (CPO, mPWR) is the product of mean arterial pressure and cardiac output. However, this does not account for the pulsatility of the circulation. Continuous, synchronized measurement of the proximal aortic flow and aortic pressure enables calculation of total hydraulic power (tPWR) (Nicolaas Westerhof et al., 2019), as illustrated in **Figure 2B**.

The difference between tPWR and mPWR is called *oscillatory power* (oPWR), and the ratio between oPWR and tPWR yields the *oscillatory power fraction* (OPF) (Nicolaas Westerhof et al., 2019).

*Oscillatory power* is considered the energy necessary to generate and deliver pulsatile blood flow and, hence, *OPF* has been proposed as a measure of coupling (Cholley and Le Gall, 2016).

Our research group has developed a system for synchronized measurement of invasive blood pressure and ultrasound-derived cardiac output, enabling estimation of both total, mean, and oscillatory power at the bedside.

OPF is potentially more easily applicable at the bedside by measuring flow/cardiac output and invasive arterial blood pressure than by using cumbersome approximation of Ea and Ees, particularly Ees, which is estimated using methods that have not been validated in cohorts of intensive care pathology and generally demonstrate limited accuracy (Wo et al., 2019).

However, the relationship between power-based and Ea/Ees indices across a broad range of physiological conditions remains poorly characterized.

Therefore, the aim of this study was to examine the relationship between the invasively measured “gold-standard” elastance ratio Ea/Ees and OPF in pharmacologically induced extreme cardiovascular conditions in a well-established porcine model.

## 2 Methods

### 2.1 Animals and ethical approval

**Figure 3:**
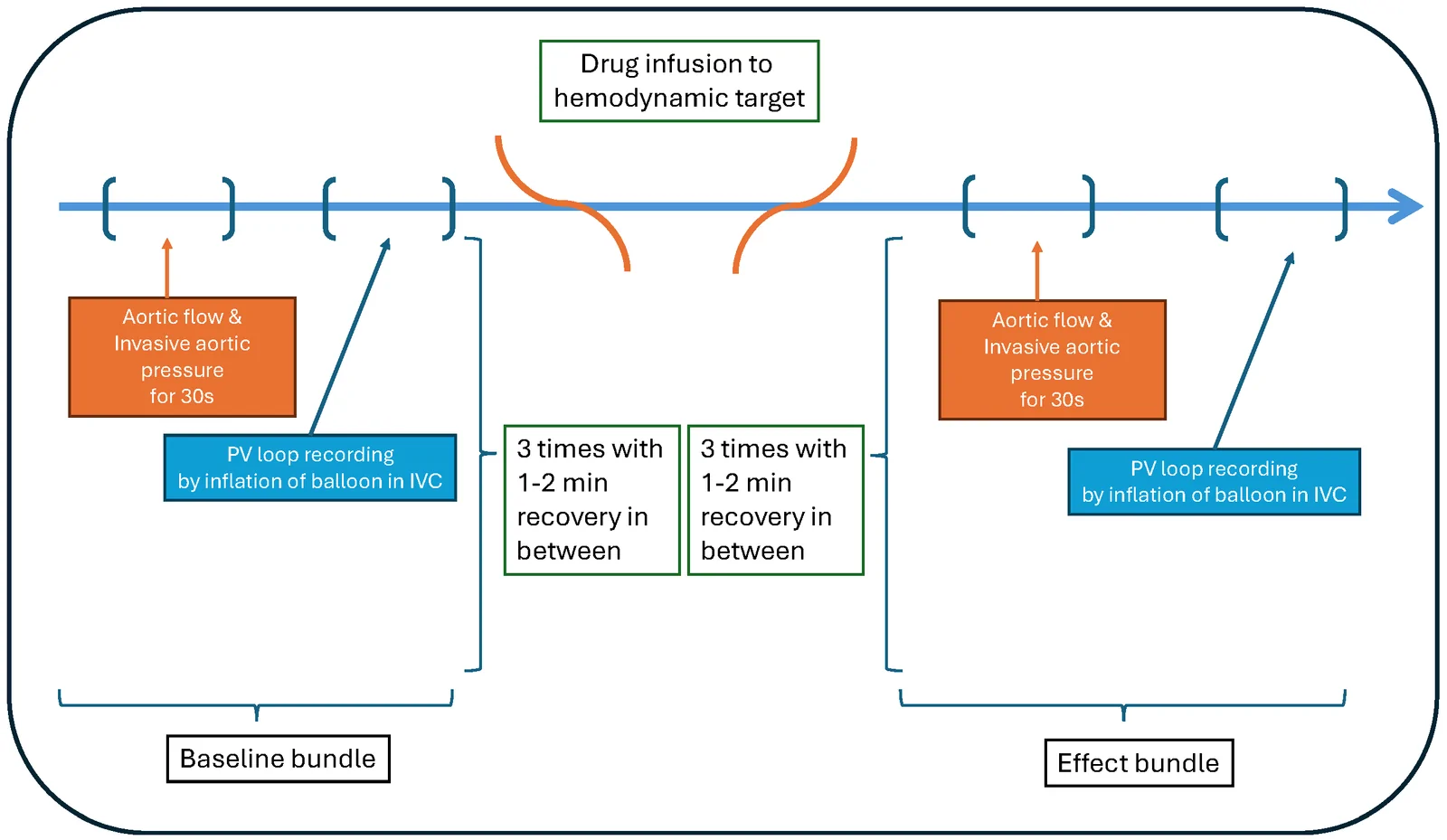
Experimental protocol for drug interventions. For each of the four drugs, a dedicated baseline was obtained immediately before infusion to serve as its own control. All baseline and drug recordings covered at least three respiratory cycles; no respiratory hold was required due to the open thorax. Baseline bundles consisted of 30 s simultaneous recordings of aortic flow and aortic pressure for power calculations, followed by acquisition of the end-systolic pressure–volume relationship (ESPVR) during transient inferior vena cava balloon inflation to acutely reduce preload. This baseline bundle (power + PV loops) was repeated three times, separated by 1–2 min. Each drug was then infused via a central venous catheter to predefined hemodynamic targets. At target, three effect bundles were recorded as above, followed by a new set of three baseline bundles before the next drug, yielding six bundles per drug and 24 measurements per animal.

The experimental protocol (**Figure 3**; protocol in the **Supplementary Material**) was approved by the Norwegian Food Safety Authority (Project No. 20832: “Oscillatory Power Fraction, a parameter for ventriculoarterial coupling”). Fourteen pigs (*Sus scrofa domesticus*; five females, nine males; 40.4 kg ± 4.6 kg, range: 32.4–47 kg) were acclimatized for 4–6 days with a standardized diet, environmental enrichment, and water *ad libitum*.

#### 2.1.1 Preparation

After the sufficient effect of intramuscularly administered premedication (ketamine: 10 mg kg^−1^, xylazine: 2 mg kg^−1^, or dexmedetomidine: 80 µg kg^−1^), anesthesia was induced intravenously with fentanyl (4–8 µg kg^−1^), thiopentone (2–4 mg kg^−1^), and ketamine (4–8 mg kg^−1^) in addition to 1mg of atropine. Animals were intubated, placed supine, and mechanically ventilated (Aisys, GE Healthcare Norge AS, Oslo, Norway) in volume-controlled mode with a tidal volume of ~ 10 mL kg^−1^ and a respiratory rate adjusted to achieve an end-tidal CO_2_ of 4.5%–5.5%. A suprapubic urinary catheter was inserted.

Anesthesia was maintained by continuous intravenous infusion of ketamine (5 mg kg^−1^ h^−1^), propofol (8 mg kg^−1^ h^−1^), and dexmedetomidine (4 µg kg^−1^ h^−1^); no neuromuscular blocker was used at any time.

Ringer’s lactate (10 mL kg^−1^) was given as a bolus, followed by 12 mL kg^−1^ h^−1^ as maintenance. Amiodarone (4 mg kg^−1^ iv) was administered once prior to sternotomy, and heparin (2,500 IU iv) was given after sternotomy and repeated hourly.

Rectal temperature was maintained at 38°C with a heating blanket. A diathermy neutral electrode was placed over the right gluteal muscle.

#### 2.1.2 Instrumentation

Central venous access was obtained via the left internal jugular vein. Arterial pressure was monitored using a 6 Fr introducer in the right carotid artery (strain gauge), and a high-fidelity pressure catheter (Micro-Cath, Millar SPR-350, Houston, TX, USA) introduced via the right femoral artery (Terumo, Elkton, MD, USA) and positioned in the descending or abdominal aorta.

A Fogarty arterial embolectomy catheter (Edwards Lifesciences, Irvine, CA, USA) was inserted via the left femoral vein and its 2 mL balloon placed in the inferior vena cava under manual palpation and ultrasound guidance.

After median sternotomy, a transit-time flowmeter (Transonic, AdInstruments, Dunedin, New Zealand) was placed around the proximal ascending aorta. A conductance pressure-volume catheter (Venticath-510, Millar/AdInstruments, Dunedin, New Zealand), calibrated in room-temperature saline, was inserted under ultrasound guidance via a 5-Fr apical introducer across the left ventricular long axis into the LV outflow tract, with the tip advanced across the aortic valve. Catheter position was optimized using echocardiography, intraventricular pressure tracings, and pressure–volume (PV) loop quality. Instantaneous pressure, flow, and PV data were recorded using a PowerLab and MPVS Ultra system (AdInstruments, Dunedin, New Zealand) with a sampling frequency of 200 Hz. The number of active PV electrodes (out of 12) was selected based on loop quality. The measurement results were compiled in in-house software to enable synchronous visualization of PV loops and waveforms.

### 2.2 Experimental protocol

The experimental protocol is illustrated in **Figure 3**. As shown, each animal was studied under four pharmacological conditions (phenylephrine, nitroprusside, esmolol, and dobutamine), each with its own immediately preceding baseline serving as the control. For every condition, we acquired baseline and drug-effect data over at least three respiratory cycles without respiratory holds, as the open thorax minimized respiratory artifacts.

At each baseline, we first recorded simultaneous aortic flow and arterial pressure for power calculations, followed by assessment of the end-systolic pressure–volume relationship (ESPVR) during transient preload reduction via inferior vena cava balloon inflation. This combined “power + PV-loop” bundle was repeated three times with short recovery intervals.

Drugs were titrated via a central venous line to predefined hemodynamic targets (see the section below for details). At target, three effect bundles were obtained, after which a new baseline series was recorded before the next drug. This protocol yielded six measurement bundles per drug and 24 per animal.

The sequences of the four drug infusions were not altered during the trial mostly because of practical reasons. Piglets have been shown to resemble pediatric humans in terms of physiological and biochemical aspects, and pharmacokinetics are considered similar (Gasthuys et al., 2016; Ayuso et al., 2020; Millecam and Devreese, 2017). There is even evidence of faster metabolism of dopamine in neonatal piglets than in human neonates (Cheung, 2018). By using defined periods of 5–10 min between different drug administrations, we assured that two to four times of the literature-described half-life (T1/2) in humans had elapsed, allowing for reasonable washout and reliable return to baseline physiology (T1/2 for sodium nitroprusside: ca. 2 min (Dhochak and Lodha, 2022); T1/2 for esmolol: 2.7–4.8 min (Wiest and Haney, 2012); T1/2 for phenylephrine: ca. 5 min; and *T* ½ dobutamine ca. 2 min (Peck et al., 2008).

At the end of the experiment, the animals were euthanized according to the approved protocol (thiopentone 100 mg kg^−1^ iv, see the protocol in the **Supplementary Material**). All surgical procedures were performed by the same experienced and certified operator (see the experimental protocol in the **Supplementary Material**).

### 2.3 Drugs used for hemodynamic challenges

All animals underwent treatment with four known drugs to induce different physiological extremes according to the protocol.

Phenylephrine is a synthetic sympathomimetic drug that acts directly on α1 receptors without affecting β-adrenoceptors. It raises systemic vascular resistance and, hence, blood pressure (Peck et al., 2008). The effect was defined as an increase in mean arterial pressure of 40% compared with baseline.

Sodium nitroprusside delivers vasodilation to arteries and veins, leading to reduced systemic vascular resistance and a resulting fall in blood pressure. Likewise, it increases venous pooling and decreases preload. It has no direct effects on contractility (Peck et al., 2008). The effect was defined as a decrease in mean arterial blood pressure of 40% compared with baseline.

Esmolol is a cardioselective β1 blocker and has no intrinsic sympathomimetic activity (Peck et al., 2008). It was considered effective when arterial d*P*/d*t* was reduced to 50% of baseline.

Dobutamine is a synthetic catecholaminergic sympathomimetic drug that acts on β1 receptors, with a small effect on β2 receptors as well. It increases contractility, heart rate, and myocardial oxygen demand (Peck et al., 2008). The effect was considered present when arterial d*P*/d*t* increased by at least 50% compared with baseline.

### 2.4 Data acquisition

#### 2.4.1 Cardiac power analysis

Pressure and flow recordings were processed with in-house-developed software, as illustrated in **Figure 2**. Aortic pressure and ventricular pressure were manually aligned and synchronized with aortic flow. The resulting signals were used to derive cardiac power indices (tPWR, mPWR, oPWR), d*P*/d*t*, and conventional hemodynamic parameters (arterial pressure, stroke volume, and cardiac output).

#### 2.4.2 Elastance calculations

For each baseline and effect state, ESPVR was derived from the initial PV loops obtained during transient preload reduction. End-systolic points from approximately 9–12 consecutive beats (≥ 3 respiratory cycles) were used to minimize compensatory responses. The slope of the linear ESPVR (Ees) was determined by linear fitting of end-systolic volume–pressure pairs using an interactive analysis tool, in accordance with the approach described by Kass et al. (1986).

**Figure 4:**
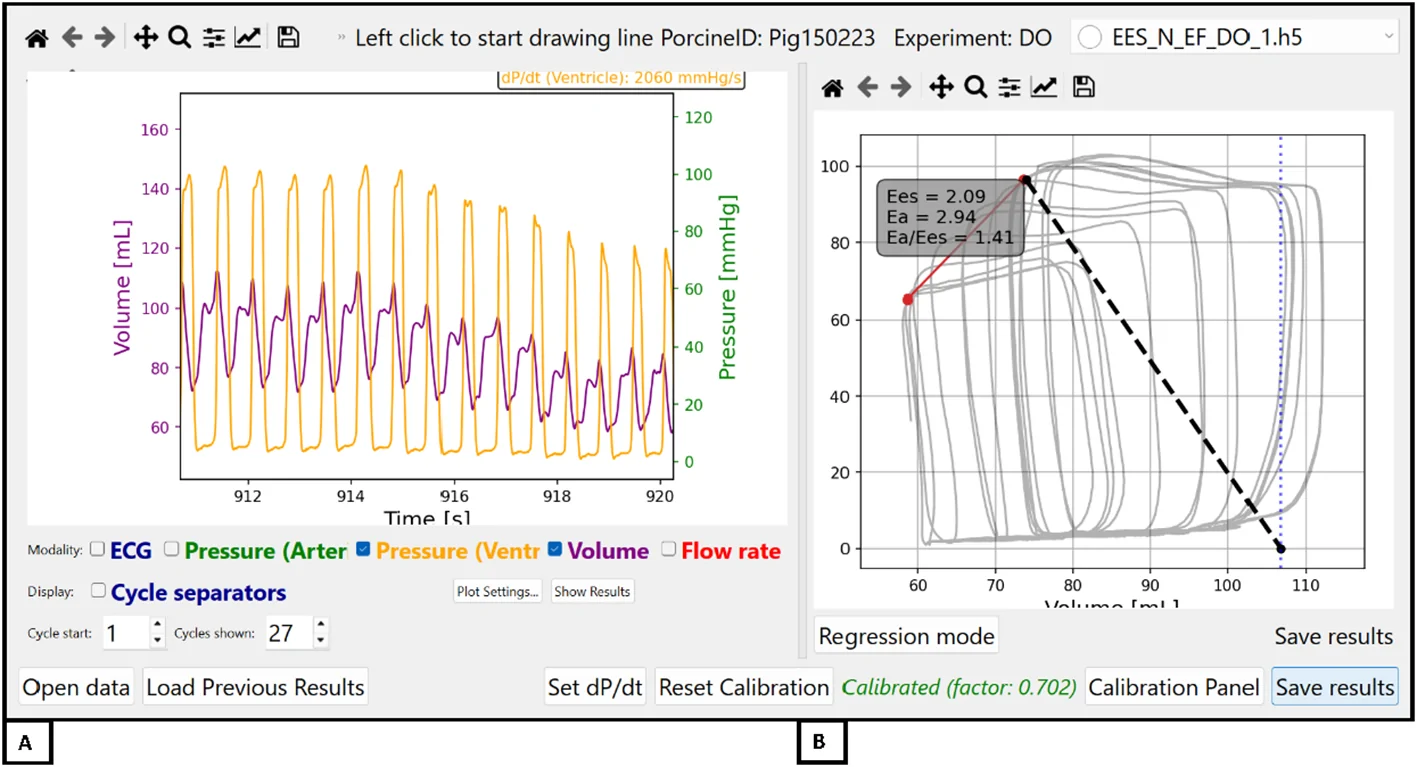
ESPVR and Ea analysis. During transient preload reduction, end-systolic points from 9 to 12 beats (≥ 3 respiratory cycles) were extracted from initial PV loops **(A)**. ESPVR slope (Ees) was obtained by linear regression of end-systolic pressure–volume pairs. Effective arterial elastance (Ea) was calculated from the line linking initial end-systolic and end-diastolic volumes **(B)**. Intraventricular volume was calibrated to aortic flow from the transit-time probe due to unreliable echocardiographic stroke volume in pigs.

Effective Ea was determined as the slope of the line connecting the initial end-systolic volume to end-diastolic volume. Echocardiographic stroke volume estimation in pigs was unreliable; therefore, intraventricular volume was calibrated against aortic flow measured by the transit-time probe placed as close as possible to the aortic valve. An example PV loop analysis is shown in **Figure 4**.

#### 2.4.3 Statistical methods

Our initial power calculation was based on detecting an association between Ea/Ees and OPF by using regression analysis and Pearson’s correlation. We assumed a power of 80% and an alpha of 0.05 to detect a correlation of *r* = 0.7. Both an interactive tool and a table for sample size estimation for correlation analysis indicated that 13 animals would be required. A total of 14 animals were ultimately included, but because of corrupted data resulting from suboptimal device settings during acquisition that affected flow and/or catheter-derived measurements, the effective sample size was reduced to 10–12.

Continuous variables are presented as means (SD). Normality of model residuals was assessed by visual inspection of *Q*–*Q* plots and evaluated using the Shapiro–Wilk test. A significance level of *p* < 0.05 was used.

The primary analysis compared baseline and effect-state measurements for each drug intervention. Given the repeated-measures design, with each animal contributing up to three baseline–effect observation pairs per drug, linear mixed-effects models were fitted with animal as a random effect to account for within-animal correlation. The effect of the intervention was evaluated using Wald *Z*-tests for the fixed effect of measurement state (baseline vs. effect). Effect sizes were quantified using paired Cohen’s *d*, calculated as the mean of within-subject differences divided by the standard deviation of those differences.

The responsiveness of each index to the drug challenges was quantified from the linear mixed-effects models described above as the percentage change from baseline and the paired effect size (Cohen’s *d*).

The association between OPF and Ea/Ees was assessed at two levels. First, for comparability with the descriptive literature, Pearson’s correlation coefficient (*r*) and the coefficient of determination (*R*^2^) were computed on animal-averaged values, separately for each drug at baseline and in the effect state. Averaging reduces the effective sample size to the number of animals (*n* = 10–12); therefore, these coefficients are reported with 95% confidence intervals (Fisher’s *z*) and interpreted as descriptive only.

Second, the coupling of drug responses, i.e., whether animals showing a larger change in Ea/Ees also showed a larger change in OPF, was assessed by correlating each animal’s baseline-to-effect change in the two indices. The replicate recordings within each state are sequential repeats and did not correspond across states; therefore, recordings were not paired by replicate index. Each animal’s change was taken as the difference of its state means, which is equivalent to averaging all possible baseline-to-effect pairings. The effective sample size for this analysis was the number of animals rather than the number of observations, as the analysis concerned between-animal variation in the magnitude of the response. Averaging replicates leaves residual measurement error in each animal’s change score, which attenuates the correlation; therefore, replicate scatter was used to estimate the reliability of the change scores, and the coefficients were additionally reported with correction for attenuation as a sensitivity analysis. This correction adjusts bias and not variance, and confidence intervals are unchanged.

As a methodological check, a repeated-measures correlation (Bakdash and Marusich, 2017) was also computed as the partial correlation between OPF and Ea/Ees after adjustment for animal and for measurement state (baseline or drug effect), both entered as categorical factors in an ordinary least-squares model, with the correlation derived from the *t*-statistic of the Ea/Ees coefficient. With each animal contributing only two measurement states, this estimand conditions out the average drug effect and therefore does not address responsiveness to the intervention. This is reported in the **Supplementary Material**.

Correlation coefficients were interpreted following Schober et al (Schober et al., 2018). as negligible (|*r*| < 0.10), weak (0.10–0.39), moderate (0.40–0.69), strong (0.70–0.89), or very strong (≥ 0.90). Correlations computed across drugs and measurement states were adjusted for multiple comparisons using the Benjamini–Hochberg procedure. In place of a *post hoc* power calculation, a sensitivity analysis reporting the smallest correlation detectable with 80% power at *α* = 0.05 is provided for each analysis strategy.

*R*^2^ was understood as the proportion of the variance of one variable (%) explained by the other. Effect size measured by Cohen’s *d* was interpreted as small (0.2–0.25), medium (0.5), or large (0.8–0.9) (Quintana, 2017; Cohen, 1992).

All statistical analyses were performed using Python version 3.11, with statsmodels for linear mixed-effects models and multiple-comparisons adjustments, and SciPy and pandas packages for correlation analyses. Figures were generated using matplotlib and seaborn.

## 3 Results

Fourteen animals (five females, nine males) underwent treatment according to protocol. Additional within-animal exclusions were performed to remove clearly faulty measurements, resulting in varying sample sizes across interventions.

For analyses of power-derived variables, the number of animals included in each drug condition was 13 for phenylephrine, 12 for sodium nitroprusside, 12 for esmolol, and 13 for dobutamine. Corresponding sample sizes for elastance-based measures (Ea/Ees) were slightly lower (*n* = 12, 11, 10, and 10, respectively) due to additional exclusions in pressure–volume loop analysis.

The evolution of hemodynamic variables for each drug challenge is shown in **Tables 1**–**4** (phenylephrine, sodium nitroprusside, esmolol, and dobutamine, respectively).

**Table 1:** Hemodynamic measurements, elastance calculations, and derived power metrics during phenylephrine challenge.

Parameter	Before	After	% Change	Cohen’s *d*	p(ME)
Ees (mmHg·mL^−1^)	1.17 (0.35)	1.60 (0.76)	+ 36.7%	0.741	< 0.001
Ea (mmHg·mL^−1^)	2.58 (0.55)	3.44 (0.81)	+ 33.4%	1.732	< 0.001
VAC (Ea/Ees)	2.46 (0.91)	2.71 (1.31)	+ 10.5%	0.283	0.120
Total power (W)	0.55 (0.18)	0.80 (0.34)	+ 45.5%	1.337	< 0.001
Mean power (W)	0.45 (0.15)	0.65 (0.28)	+ 46.7%	1.333	< 0.001
Oscillatory power (W)	0.11 (0.04)	0.15 (0.06)	+ 40.5%	1.071	< 0.001
OPF (%)	19.40 (2.83)	19.10 (2.95)	− 1.5%	− 0.097	0.578
MAP (mmHg)	75.88 (13.41)	99.21 (19.28)	+ 30.8%	3.210	< 0.001
d*P*/d*t*_max_ ventricle (mmHg·s^−1^)	1,120.26 (248.98)	1,474.58 (439.85)	+ 31.6%	1.371	< 0.001
SystolicBP aorta (mmHg)	104.30 (15.77)	133.85 (22.04)	+ 28.3%	3.946	< 0.001
DiastolicBP aorta (mmHg)	59.49 (12.45)	77.51 (18.29)	+ 30.3%	2.419	< 0.001
CO (L·min^−1^)	2.60 (0.58)	2.91 (0.82)	+ 11.6%	0.645	< 0.001
SV flow (mL)	38.84 (9.88)	40.45 (10.65)	+ 4.2%	0.354	0.019
HR (beats·min^1^	68.57 (11.80)	72.04 (10.79)	+ 5.1%	0.368	0.006

Values are presented as means (SD). The before–after change was calculated using a Wald *Z*-test. Paired Cohen’s *d* was calculated to quantify the effect size (small effect: 0.2, medium effect: 0.5, large effect: 0.8). Mixed-effects models were applied with a significance level of *p*(ME) < 0.05.

*Ees*, endsystolic elastance; *Ea*, arterial elastance; *VAC*, ventriculoarterial coupling as ratio of Ea/Ees; *OPF*, oscillatory power fraction; *MAP*, mean arterial pressure; *dP/dt_max_*, peak rate of pressure change over time (contractility); *CO*, cardiac output; *SV*, stroke volume; *HR*, heart rate.

**Table 2:** Hemodynamic measurements, elastance calculations, and derived power metrics during sodium nitroprusside challenge.

Parameter	Before	After	% Change	Cohen’s *d*	*p*(ME)
Ees (mmHg·mL^−1^)	1.32 (0.61)	1.47 (0.57)	+ 11.2%	0.219	0.304
Ea (mmHg·mL^−1^)	3.31 (1.57)	2.48 (0.94)	− 25.1%	− 0.761	< 0.001
VAC (Ea/Ees)	2.77 (1.12)	1.77 (0.52)	− 36.2%	− 1.239	< 0.001
Total power (W)	0.59 (0.21)	0.29 (0.09)	− 50.8%	− 2.004	< 0.001
Mean power (W)	0.48 (0.17)	0.24 (0.07)	− 50.0%	− 1.935	< 0.001
Oscillatory power (W)	0.12 (0.04)	0.05 (0.02)	− 53.7%	− 2.083	< 0.001
OPF (%)	20.09 (3.27)	18.43 (4.66)	− 8.3%	− 0.453	0.004
MAP (mmHg)	77.26 (11.35)	44.10 (7.54)	− 42.9%	− 3.372	< 0.001
d*P*/d*t*_max_ ventricle (mmHg·s^−1^)	1,097.97 (238.88)	986.82 (368.23)	− 10.1%	− 0.419	0.010
SystolicBP aorta (mmHg)	106.97 (12.76)	60.07 (11.27)	− 43.8%	− 3.451	< 0.001
DiastolicBP aorta (mmHg)	59.06 (9.86)	34.60 (6.54)	− 41.4%	− 3.105	< 0.001
CO (L·min^−1^)	2.71 (0.68)	2.41 (0.58)	− 11.1%	− 0.934	< 0.001
SV flow (mL)	37.42 (11.00)	28.23 (8.31)	− 24.6%	− 1.604	< 0.001
HR (beats·min^−1^)	74.09 (12.46)	88.06 (18.14)	+ 18.9%	0.954	< 0.001

Values are presented as means (SD). Before–after changes were assessed using the Wald *Z*-test. Paired Cohen’s *d* was calculated to quantify the effect size (small effect, 0.2; medium effect, 0.5; large effect, 0.8). A mixed-effects model was applied with a significance level of *p* < 0.05.

*Ees*, endsystolic elastance; *Ea*, arterial elastance; *VAC*, ventriculoarterial coupling as ratio of Ea/Ees; *OPF*, oscillatory power fraction; *MAP*, mean arterial pressure; *dP/dt_max_*, peak rate of pressure change over time (contractility); *CO*, cardiac output; *SV*, stroke volume; *HR*, heart rate.

**Table 3:** Hemodynamic measurements, elastance calculations, and derived power metrics during the esmolol challenge.

Parameter	Before	After	% Change	Cohen’s *d*	*p*(ME)
Ees (mmHg·mL^−1^)	1.25 (0.50)	0.84 (0.27)	− 32.4%	− 1.090	< 0.001
Ea (mmHg·mL^−1^)	2.75 (0.62)	2.58 (0.51)	− 6.3%	− 0.475	0.103
VAC (Ea/Ees)	2.48 (0.78)	3.36 (1.42)	+ 35.4%	0.799	< 0.001
Total power (W)	0.63 (0.23)	0.47 (0.20)	− 25.0%	− 1.982	< 0.001
Mean power (W)	0.51 (0.19)	0.37 (0.17)	− 26.6%	− 2.031	< 0.001
Oscillatory power (W)	0.12 (0.04)	0.10 (0.03)	− 18.4%	− 1.364	< 0.001
OPF (%)	19.04 (3.10)	21.36 (4.28)	+ 12.2%	1.254	< 0.001
MAP (mmHg)	80.97 (13.63)	63.75 (16.78)	− 21.3%	− 2.335	< 0.001
d*P*/d*t*_max_ ventricle (mmHg·s^−1^)	1,168.72 (354.80)	741.87 (190.92)	− 36.5%	− 1.523	< 0.001
SystolicBP aorta (mmHg)	109.97 (16.16)	89.31 (19.61)	− 18.8%	− 2.400	< 0.001
DiastolicBP aorta (mmHg)	64.09 (12.65)	49.75 (15.31)	− 22.4%	− 2.142	< 0.001
CO (L·min^−1^)	2.79 (0.84)	2.53 (0.60)	− 9.1%	− 1.200	< 0.001
SV flow (mL)	38.17 (11.38)	33.76 (6.60)	− 11.6%	− 2.043	< 0.001
HR (beats·min^−1^)	75.09 (14.71)	75.10 (10.30)	+ 0.0%	0.300	0.028

Values are presented as means (SD). Before–after changes were assessed using the Wald *Z*-test. Paired Cohen’s *d* was calculated to quantify effect size (small effect, 0.2; medium effect, 0.5; large effect, 0.8). A mixed-effects model was applied with a significance level of *p* < 0.05.

*Ees*, endsystolic elastance; *Ea*, arterial elastance; *VAC*, ventriculoarterial coupling as ratio of Ea/Ees; *OPF*, oscillatory power fraction; *MAP*, mean arterial pressure; *dP/dt_max_*, peak rate of pressure change over time (contractility); *CO*, cardiac output; *SV*, stroke volume; *HR*, heart rate.

**Table 4:** Hemodynamic measurements, elastance calculations, and derived power metrics during the dobutamin challenge.

Parameter	Before	After	% Change	Cohen’s *d*	*p*(ME)
Ees (mmHg·mL^−1^)	1.52 (0.56)	2.01 (1.14)	+ 32.1%	0.487	< 0.001
Ea (mmHg·mL^−1^)	2.80 (0.66)	3.17 (1.22)	+ 13.3%	0.421	0.011
VAC (Ea/Ees)	2.10 (0.85)	1.81 (0.61)	− 13.5%	− 0.364	0.010
Total power (W)	0.61 (0.24)	0.82 (0.29)	+ 34.1%	1.627	< 0.001
Mean power (W)	0.49 (0.21)	0.65 (0.23)	+ 33.9%	1.550	< 0.001
Oscillatory power (W)	0.12 (0.04)	0.16 (0.06)	+ 34.7%	1.518	< 0.001
OPF (%)	20.47 (4.28)	19.95 (2.77)	− 2.5%	− 0.178	0.206
MAP (mmHg)	75.91 (16.86)	83.88 (11.12)	+ 10.5%	0.541	< 0.001
d*P*/d*t*_max_ ventricle (mmHg·s^−1^)	1,155.77 (346.36)	2,425.42 (891.93)	+ 109.9%	1.667	< 0.001
SystolicBP aorta (mmHg)	104.05 (20.16)	112.71 (13.43)	+ 8.3%	0.478	< 0.001
DiastolicBP aorta (mmHg)	59.27 (15.34)	65.16 (10.74)	+ 9.9%	0.476	< 0.001
CO (L·min^−1^)	2.83 (0.83)	3.50 (1.09)	+ 23.7%	1.707	< 0.001
SV flow (mL)	35.21 (10.90)	34.34 (10.18)	− 2.5%	− 0.177	0.391
HR (beats·min^−1^)	79.97 (17.09)	103.70 (16.70)	+ 29.7%	2.665	< 0.001

Values are presented as means (SD). Before–after changes were assessed using the Wald *Z*-test. Paired Cohen’s *d* was calculated to quantify effect size (small effect, 0.2; medium effect, 0.5; large effect, 0.8). A mixed-effects model was applied with a significance level of *p* < 0.05.

*Ees*, endsystolic elastance; *Ea*, arterial elastance; *VAC*, ventriculoarterial coupling as ratio of Ea/Ees; *OPF*, oscillatory power fraction; *MAP*, mean arterial pressure; *dP/dt_max_*, peak rate of pressure change over time (contractility); *CO*, cardiac output; *SV*, stroke volume; *HR*, heart rate.

### 3.1 Phenylephrine

Phenylephrine infusion increased MAP by approximately 31% (target 40%), accompanied by increases in arterial elastance Ea (+ 33.4%, *d* = 1.7, *p*(ME) < 0.001) and ventricular elastance Ees (+ 37%, *d* = 0.741, *p*(ME) < 0.001). Cardiac output increased modestly (12%, *d* = 0.645, *p*(ME) < 0.001), mainly due to increases in both heart rate and stroke volume, consistent with increased contractility as reflected by an increase in d*P*/d*t* (32%, *d* = 1.37, *p*(ME) < 0.001). Total, mean, and oscillatory power all increased by approximately 46%, 47%, and 41%, respectively (*d* = 1.0–1.3, *p* < 0.001), whereas oscillatory power fraction (OPF, − 1.5%, *d* = − 0.097, *p*(ME) = 0.578) and ventriculoarterial coupling by Ea/Ees (+ 10.5%, *d* = 0.28, *p*(ME) = 0.120) did not change significantly.

**Figure 5:**
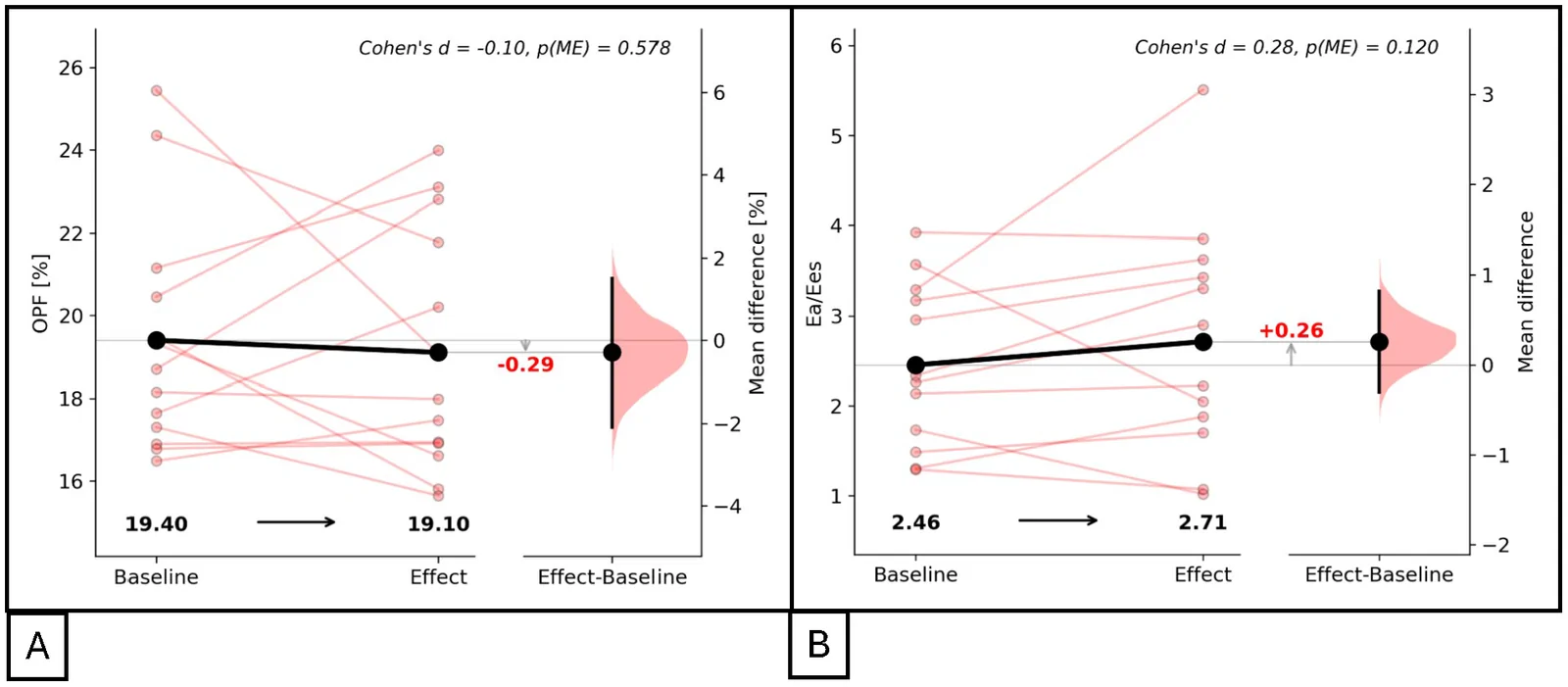
Gartner–Altman plot illustrating no significant difference in means for either OPF **(A)** or VAC **(B)** between baseline and effect measurements during phenylephrine stimulation. Cohen’s *d*, standardized effect size measure (difference between means); *p*(ME), significance level in mixed-effect model: 0.2 (small), 0.5 (medium), 0.8–0.9 (large); OPF, oscillatory power fraction; Ea/Ees, ventriculoarterial coupling.

Effect size analysis could not demonstrate a significant alteration in the means of either OPF or VAC during phenylephrine stimulation, as illustrated in **Figure 5**.

**Figure 6:**
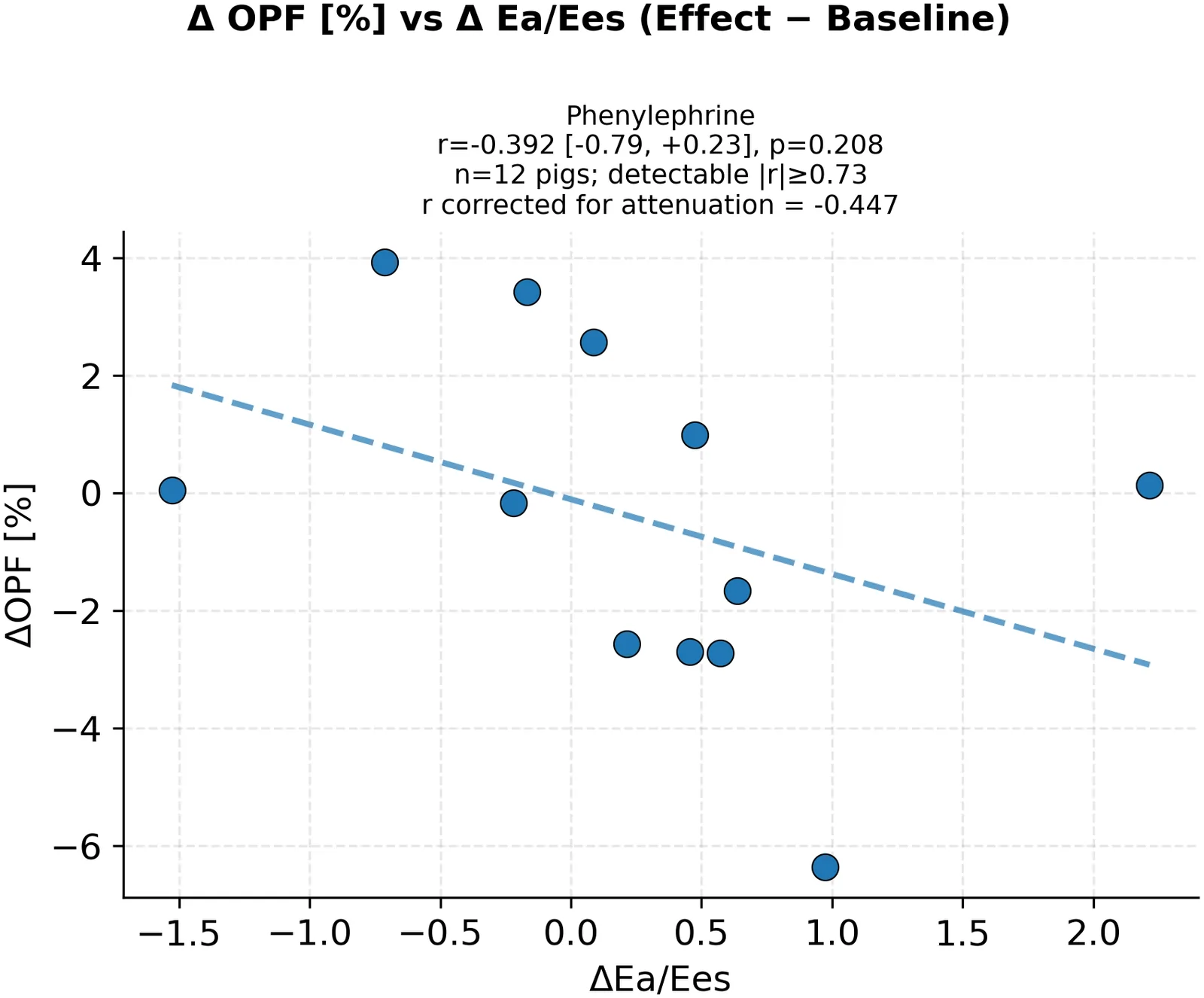
Correlation plot of the change in OPF and VAC as a function of Ea/Ees under phenylephrine stimulation. OPF, oscillatory power fraction; Eea/Ees, ventriculoarterial coupling by ratio of elastances; *r*, Pearson’s correlation coefficient; *R*^2^, coefficient of determination; *p*, significance level.

As shown in **Figure 6**, the change in OPF was not significantly associated with the change in Ea/Ees VAC and OPF during phenylephrine administration (*r* = − 0.392, 95% CI: − 0.79 to + 0.23, *p* = 0.208). With 12 animals, only correlations of |*r*| > 0.73 were detectable.

### 3.2 Sodium nitroprusside

Sodium nitroprusside reduced MAP by 43% (*d* = − 3.37, *p*(ME) < 0.001) as intended by protocol, accompanied by a marked decrease in Ea (− 25%, *d* = − 0.76, *p*(ME) < 0.001), while Ees showed a nonsignificant increase (11%, *d* = 0.21, *p*(ME) = 0.3). Contractility declined, as reflected by both ventricular d*P*/d*t*_max_ (−10.0%, *d* = − 0.42, *p*(ME) < 0.001) and a decrease in CO (− 11% (*d* = − 0.93, *p*(ME) < 0.001), the latter primarily driven by reduced stroke volume (− 25%, *d* = − 1.6, *p*(ME) < 0.001) that was not offset by a compensatory rise in heart rate (+ 19%, *d* = 0.95, *p*(ME) < 0.001). Total, mean, and oscillatory power decreased approximately by 50% (*d* ≈ − 2, all *p*(ME) < 0.001). Accordingly, VAC (Ea/Ees) decreased by 36% (*d* = − 1.24, *p*(ME) < 0.001), while OPF showed only a small, albeit statistically significant reduction (− 8.3%, *d* = − 0.44, *p*(ME) = 0.004).

**Figure 7:**
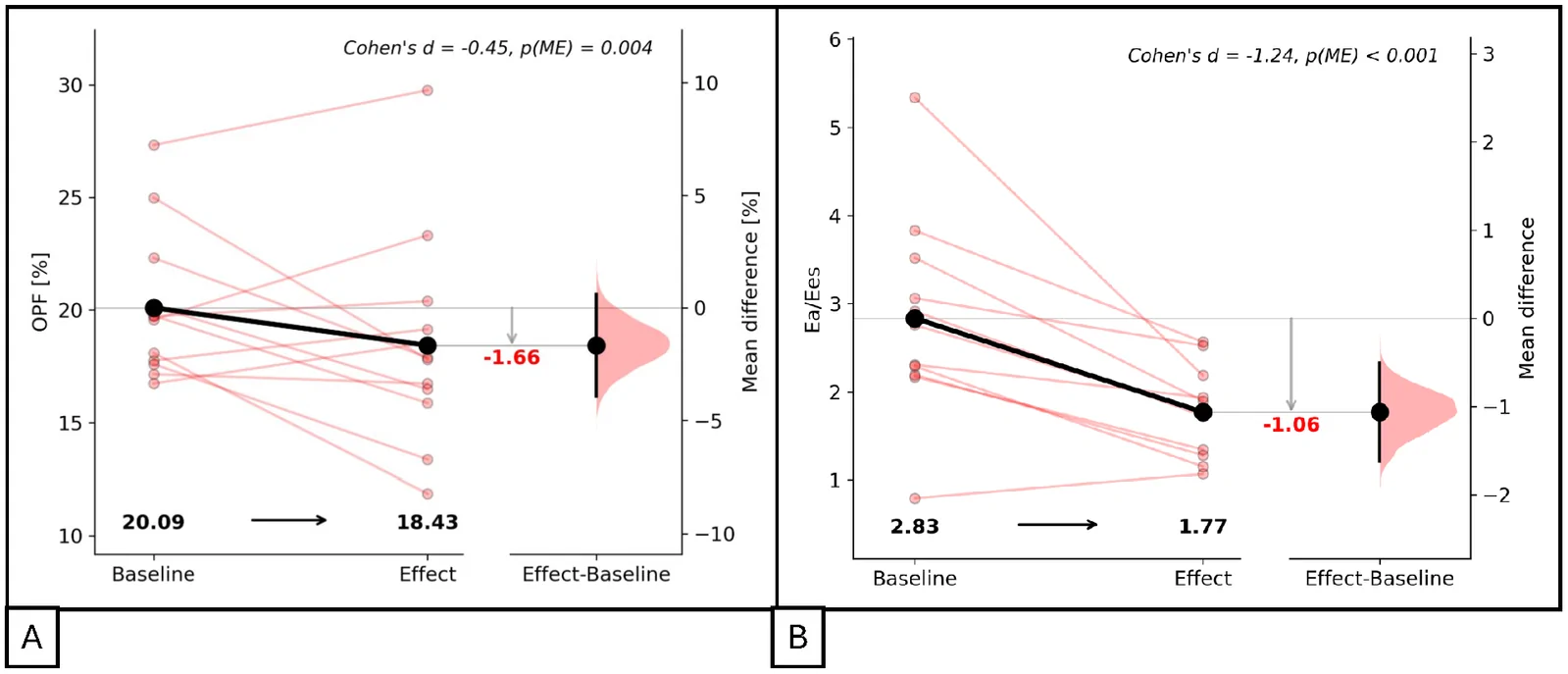
Gartner–Altman plot illustrating a significant difference in means for OPF **(A)** and VAC **(B)** between baseline and effect measurements during sodium nitroprusside stimulation. OPF, and hence pulsatile efficiency, shows a small to moderate effect, whereas VAC is strongly affected. Cohen’s *d*, standardized effect size measure (difference between means): 0.2 (small), 0.5 (medium), 0.8–0.9 (large); *p*(ME), significance level in mixed effect model; OPF, oscillatory power fraction; Ea/Ees, ventriculoarterial coupling.

As shown in **Figure 7**, differences between baseline and effect were statistically significant with both a large effect size in VAC (Cohen’s *d* = − 1.24, *p*(ME) < 0.001) and a small to moderate effect size in OPF measurements (Cohen’s *d* = − 0.45, *p*(ME) = 0.004).

**Figure 8:**
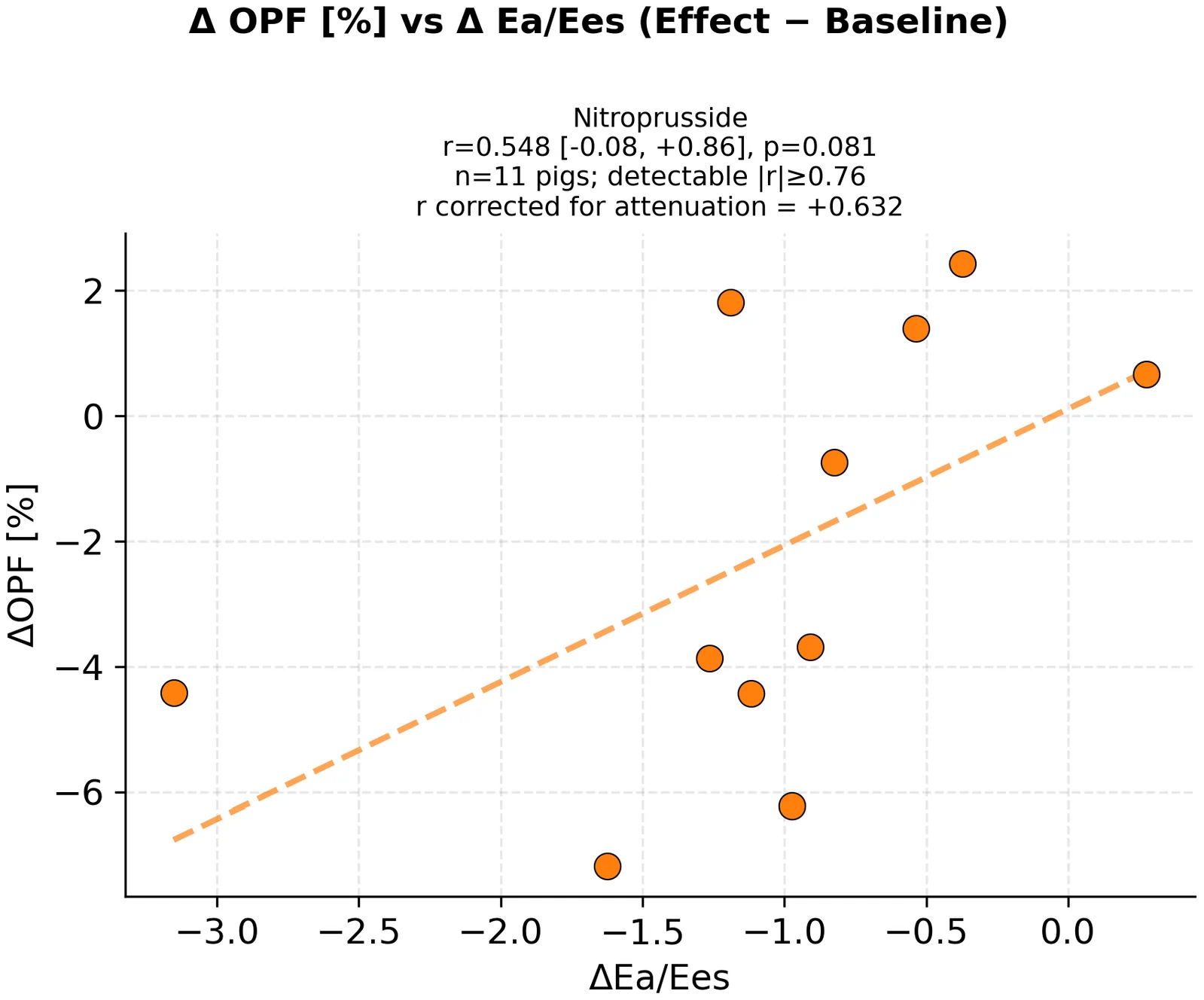
Correlation plot of OPF and VAC as a function of Ea/Ees under nitroprusside stimulation. OPF, oscillatory power fraction; Eea/Ees, ventriculoarterial coupling by ratio of elastances; *r*, Pearson’s correlation coefficient; *R*^2^, coefficient of determination; *p*, significance level.

The change in OPF was not significantly associated with the change in Ea/Ees under nitroprusside (*r* = 0.548, 95% CI: − 0.08 to + 0.86, *p* = 0.081), as illustrated in **Figure 8**. With 11 pigs, this analysis would be able to detect |*r*| > 0.76.

### 3.3 Esmolol

Esmolol reduced ventricular contractility as intended by protocol and reflected by a decrease in ventricular d*P*/d*t*_max_ (− 36.5%, *d* = − 1.52, *p*(ME) < 0.001), with a concomitant decrease in Ees (− 32.4%, *d* = − 1.09, *p*(ME) < 0.001) and a smaller nonsignificant decrease in Ea (− 6.3%, *d* = − 0.48, *p*(ME) = 0.103). Consequently, VAC (Ea/Ees) increased by 35% (*d* = 0.8, *p*(ME) <0.001). Stroke volume decreased (− 12%, *d* = − 2, *p*(ME) < 0.001), resulting in reduced CO (− 9%, *d* = − 1.2, *p*(ME) < 0.001), while heart rate remained unchanged (0.0%, *d* = 0.3, *p*(ME) = 0.028). Total, mean, and oscillatory power declined significantly by 25%, 27%, and 18%, respectively (*d* = − 2.0, − 2.0, − 1.4, all *p*(ME) < 0.001), yielding an increased OPF by 12.2% (*d* = 1.25, *p*(ME) < 0.001).

**Figure 9:**
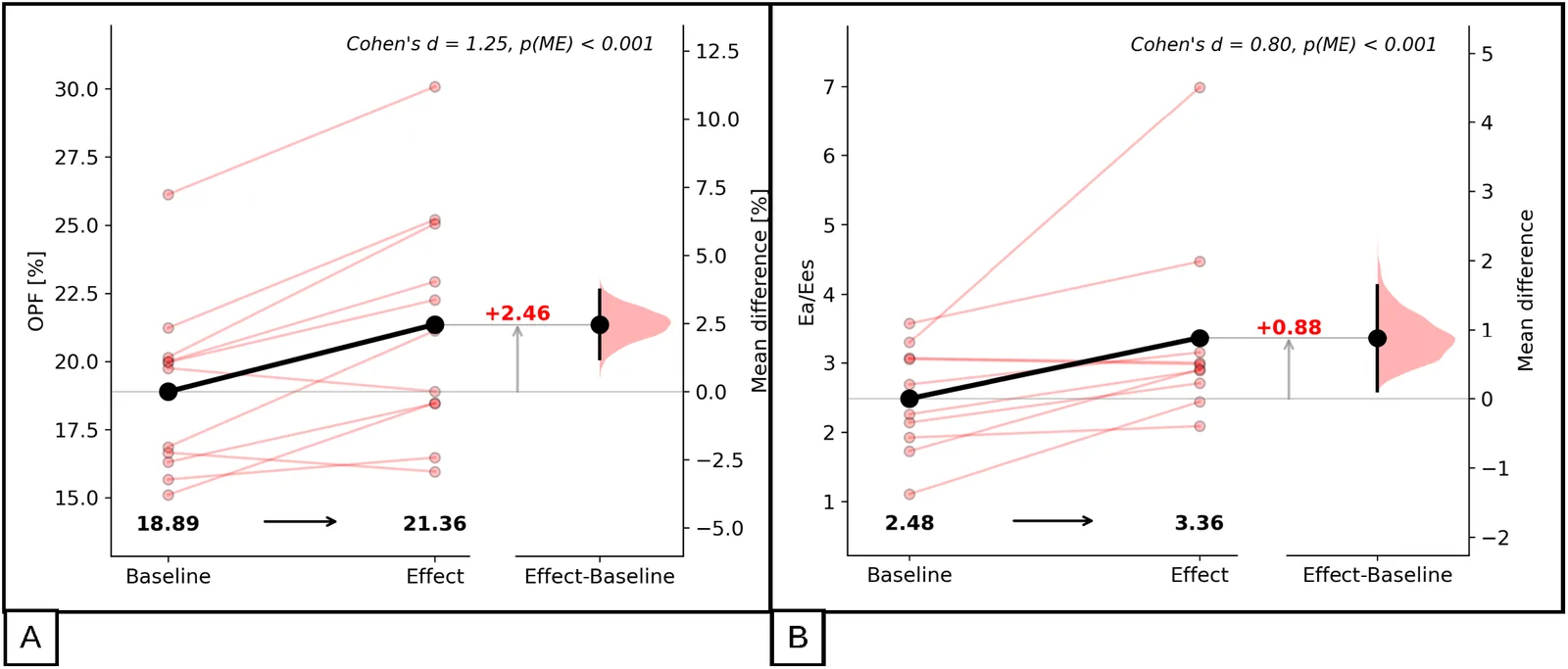
Gartner–Altman plot illustrating a significant difference in means for OPF and VAC between baseline and effect measurements during esmolol stimulation. OPF, hence pulsatile efficiency, shows a large effect **(A)**, whereas VAC is less affected **(B)**. Cohen’s *d*, standardized effect size measure (difference between means): 0.2 (small), 0.5 (medium), 0.8–0.9 (large); *p*(ME), significance level in mixed effect model; OPF, oscillatory power fraction; Ea/Ees, ventriculoarterial coupling.

As shown in **Figure 9**, effect size measurements revealed statistically significant differences between baseline and effect for both OPF and VAC measurements with a large effect (Cohen’s *d* = 1.25, *p*(ME) < 0.001 and Cohen’s *d* = 0.8, *p*(ME) < 0.001, respectively).

**Figure 10:**
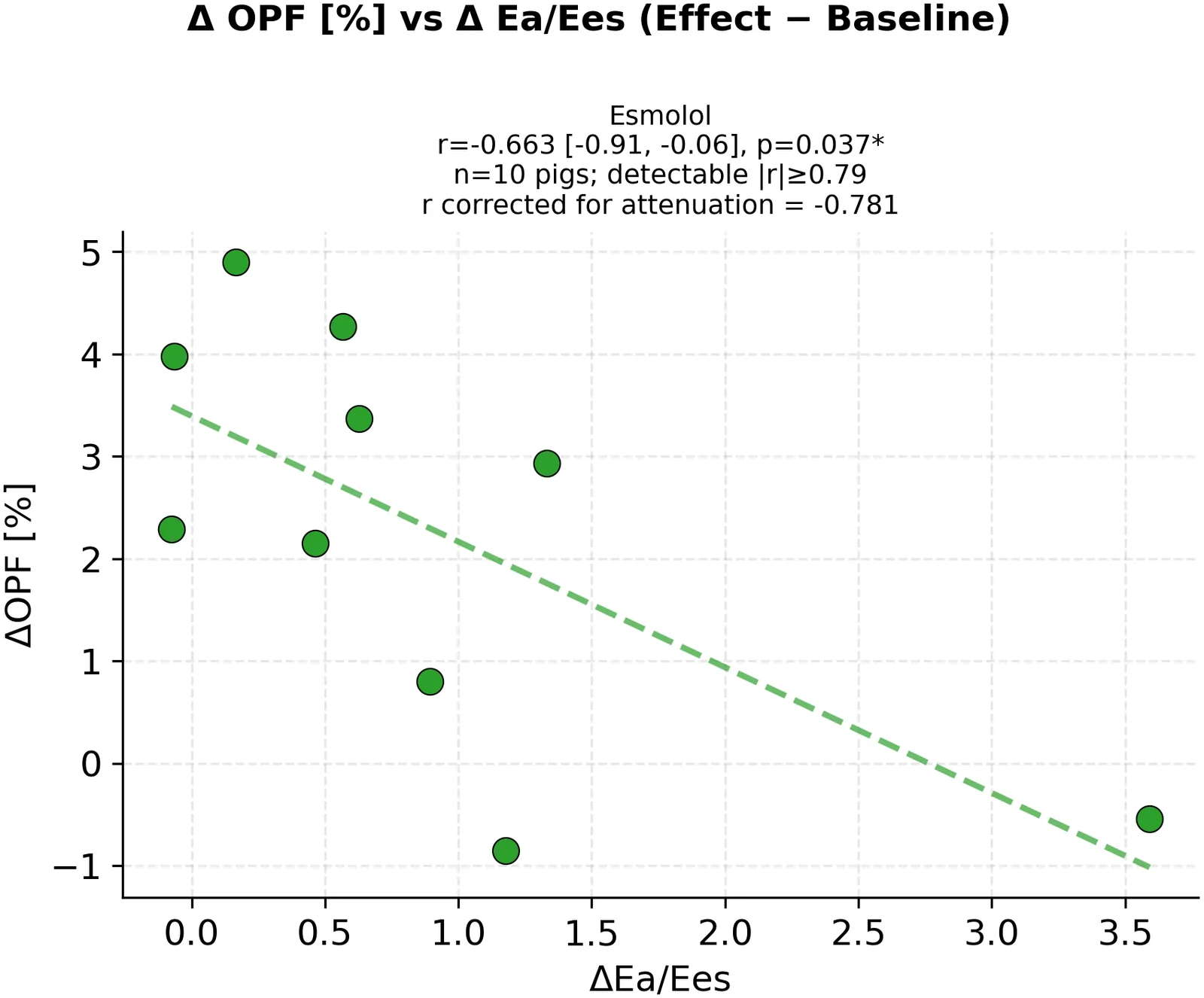
Correlation plot of the change in OPF and VAC as a function of Ea/Ees under esmolol stimulation. OPF, oscillatory power fraction; Eea/Ees, ventriculoarterial coupling calculated as the ratio of elastances; *r*, Pearson’s correlation coefficient; *R*^2^, coefficient of determination; *p*, significance level.

As illustrated in **Figure 10**, esmolol showed a moderate negative association between VAC and OPF (*r* = − 0.663, 95% CI: − 0.91 to − 0.06, *p* = 0.037). Animals with the larger rise in Ea/Ees showed a smaller rise in OPF. This did not survive adjustment for multiple comparisons and is presented as hypothesis-generating.

### 3.4 Dobutamine

Dobutamine increased heart rate by 30% (*d* = 2.7, *p*(ME) < 0.001), while stroke volume remained stable, resulting in higher CO (+ 24%, *d* = 1.7, *p*(ME) < 0.001). MAP increased modestly (+ 10.5%, *d* = 0.54, *p*(ME) < 0.001). Contractility markedly increased as reflected by the increase in ventricular d*P*/d*t*_max_ (+ 110%, *d* = 1.67, *p*(ME) < 0.001), accompanied by an increase in Ees (+ 32.6%, *d* = 0.49, *p*(ME) < 0.001), while Ea showed a smaller but still statistically significant increase (+ 13%, *d* = 0.42, *p*(ME) = 0.011). Consequently, VAC (Ea/Ees) decreased by 13.5% (*d* = − 0.36, *p*(ME) = 0.01). Total, mean, and oscillatory power rose by 34%, 34%, and 35%, respectively (*d* = 1.63, 1.55, 1.52, all *p*(ME) < 0.001), whereas OPF remained unchanged (− 2.5%, *d* = − 0.18, *p*(ME) = 0.2).

**Figure 11:**
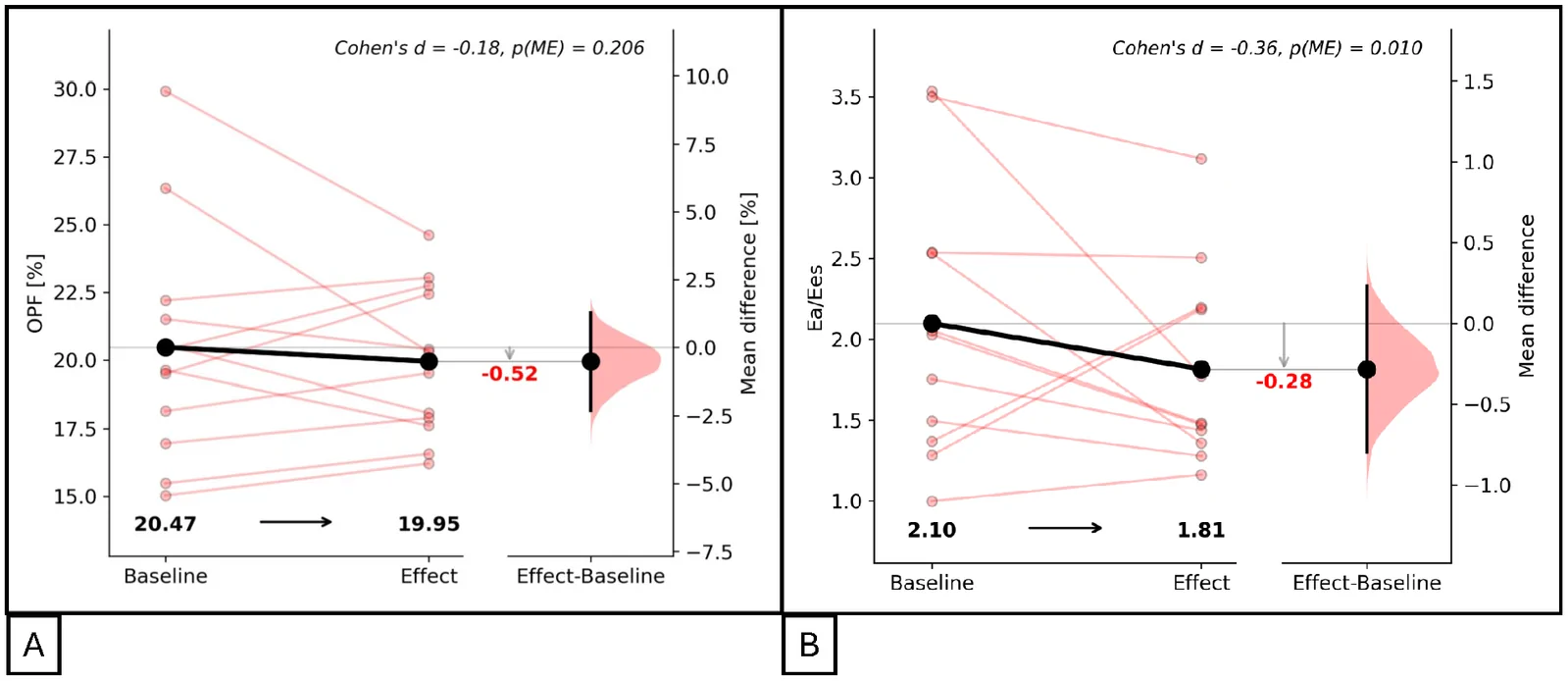
Gartner–Altman plot illustrating differences in means for OPF and VAC between baseline and effect measurements during dobutamine stimulation. OPF, hence pulsatile efficiency, shows no demonstrable effect **(A)**, whereas VAC is minimally affected **(B)**; Cohen’s *d*, standardized effect size measure (difference between means): 0.2 (small), 0.5 (medium), 0.8–0.9 (large); *p*(ME), significance level in mixed effect model; OPF, oscillatory power fraction; Ea/Ees, ventriculoarterial coupling.

According to the aforementioned results, effect size analysis demonstrated a statistically significant difference with a moderate to large effect between baseline and effect for VAC during dobutamine stimulation (Cohen’s *d* = − 0.36, *p*(ME) = 0.01) but failed to show a relevant difference for OPF (Cohen’s *d* = − 0.18, *p*(ME) = 0.206), as shown in **Figure 11**.

**Figure 12:**
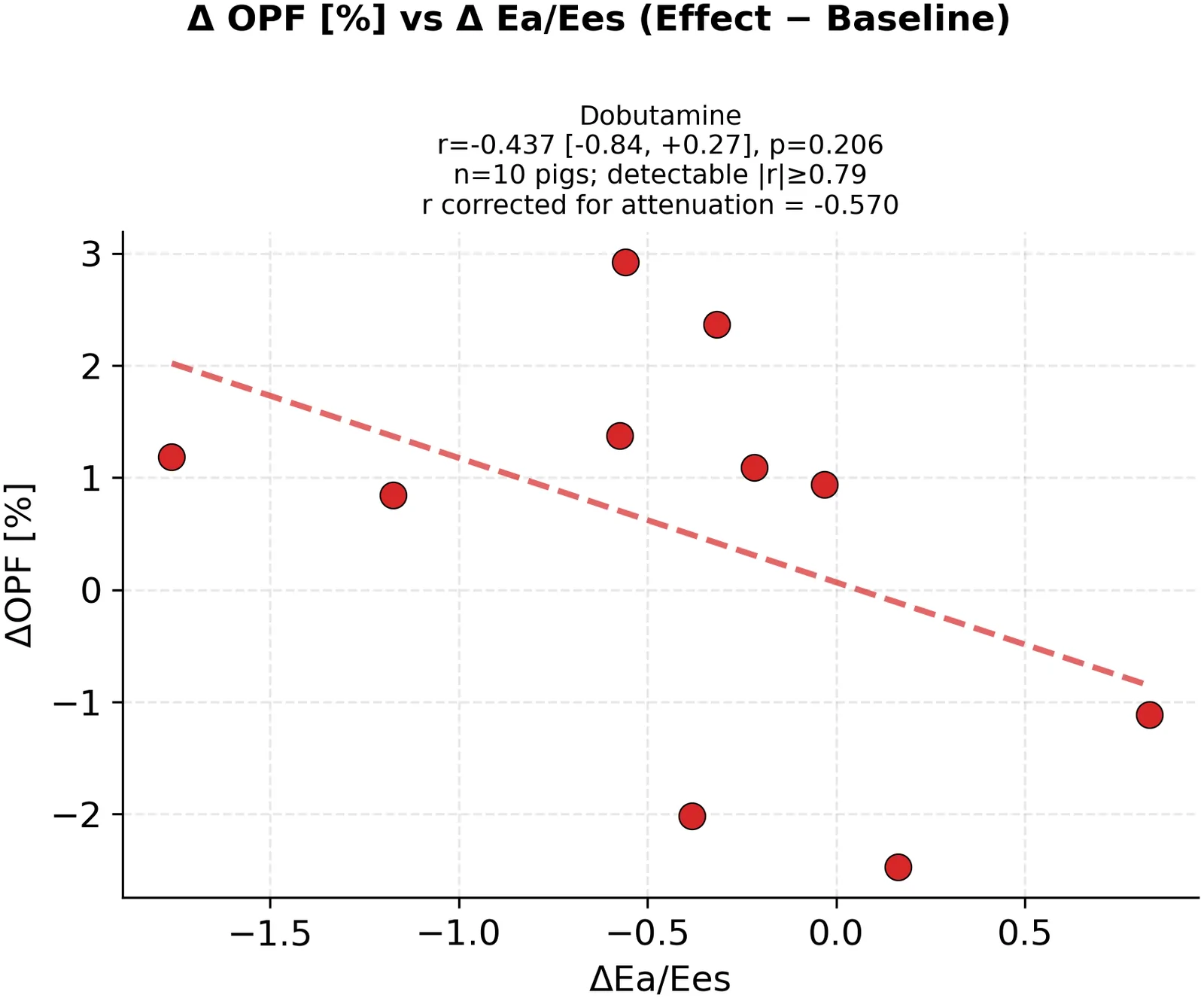
Correlation plot of the change in OPF and VAC as a function of Ea/Ees under dobutamine stimulation. OPF, oscillatory power fraction; Eea/Ees, ventriculoarterial coupling calculated as the ratio of elastances; *r*, Pearson’s correlation coefficient; *R*^2^, coefficient of determination; *p*, significance level.

Under dobutamine, the change in OPF was not significantly associated with the change in Ea/Ees (*r* = − 0.443, 95% CI: − 0.84 to + 0.27, *p* = 0.206), with the analysis able to detect only |*r*| > 0.79 (**Figure 12**).

### 3.5 Association between OPF and Ea/Ees

Across the four interventions, Ea/Ees changed by 10.5%–36.2%, whereas OPF changed by 1.5%–12.2% (**Tables 1**–**4**). Under phenylephrine and dobutamine, the change in OPF was negligible (|*d*| = 0.10 and 0.18, *p*(ME) = 0.578 and 0.206), although Ea/Ees changed measurably. Only under esmolol did both indices change substantially in the same direction.

Whether the magnitudes of the two responses were coupled, i.e., whether animals showing a larger change in Ea/Ees also showed a larger change in OPF, was assessed from within-animal change scores (**Figures 6**, **8**, **10**, **12**; **Table 5**). Coefficients ranged from *r* = − 0.66 under esmolol to *r* = + 0.55 under nitroprusside and therefore included a change of sign, but the confidence intervals were wide, and no coefficient survived adjustment for multiple comparisons. Using the replicate scatter to estimate the measurement error in each animal’s change score, the reliability of those scores was 0.75–0.93. Correcting for the resulting attenuation gives coefficients of − 0.78 to + 0.63. With 10–12 animals per intervention, correlations with |*r*| ≥ 0.73–0.79 were not detectable at 80% power.

**Table 5:** Association between the drug-induced change in OPF and the change in Ea/Ees by intervention.

Drug	*n*	*r* [95% CI]	*p*-value	Detectable |*r*|	Reliability ΔEa/Ees (ΔOPF)	*r* corrected
Phenylephrine	12	− 0.392 [− 0.79, + 0.23]	0.208	0.73	0.84 (0.92)	− 0.447
Nitroprusside	11	+ 0.548 [− 0.08, + 0.86]	0.081	0.76	0.81 (0.93)	+ 0.632
Esmolol	10	− 0.663 [− 0.91, − 0.06]	0.037	0.79	0.88 (0.82)	− 0.781
Dobutamine	10	− 0.437 [− 0.84, + 0.27]	0.206	0.79	0.75 (0.79)	− 0.570

Each animal contributes one change score per intervention, obtained as the difference between its mean drug-effect and mean baseline values, so the number of animals is the effective sample size. Reliability is the proportion of the variance in each change score attributable to true between-animal differences rather than measurement error, estimated from the replicate recordings. The *r* corrected is the correlation after adjustment for the attenuation caused by measurement error. Detectable |*r*| is the smallest correlation the analysis could have identified with 80% power at *α* = 0.05.

*n*, number of animals; *OPF*, oscillatory power fraction; *Ea/Ees*, ratio of elastances; *r*, Pearson’s correlation coefficient; *CI*, confidence interval; *p(BH)*, significance level after Benjamini–Hochberg adjustment across the 12 reported correlations.

Animal-averaged correlations at baseline and at the effect state are reported per intervention in **Sections 3.1–3.4**. Two of the 12 coefficients reached nominal significance, both under esmolol, and neither survived adjustment for multiple comparisons.

## 4 Discussion

The research question of this study was to determine whether OPF could serve as a relevant surrogate for VAC as defined by the Ea/Ees ratio. Across the pharmacologically induced hemodynamic extremes studied here, OPF did not track Ea/Ees in a manner that would permit substitution. Ea/Ees varied by 10%–36% across these challenges, while OPF varied by 1.5%–12%, and under phenylephrine and dobutamine OPF did not change detectably even though Ea/Ees did. Over the range of loading conditions achievable in this model, OPF is therefore considerably less responsive to changes in ventriculoarterial coupling than Ea/Ees. Whether the magnitudes of the two responses are coupled in individual animals remains undetermined. With 10–12 animals per intervention, the present study could only have detected correlations of |*r*| ≥ 0.73, and we regard that question as open rather than answered and the findings as hypothesis-generating. Under esmolol, the one challenge in which both indices changed substantially, the correlation between their changes was negative. Animals with the larger rise in Ea/Ees showed the smaller rise in OPF. Concordant changes at the group level therefore need not imply that the two indices track one another within individuals.

### 4.1 Phenylephrine

Phenylephrine increased arterial pressure and afterload, with parallel rises in mean and oscillatory power that left OPF largely unchanged, indicating preserved pulsatile efficiency. As Ees increased in parallel with Ea, Ea/Ees remained essentially stable, suggesting maintained ventriculoarterial matching. OPF remained relatively high (≈ 19%) compared with healthy humans (≈ 15%) and was closer to values reported in patients with suspected coronary artery disease or arterial hypertension (Nichols et al., 1986; Westerhof et al., 2019).

Earlier hypertension models using norepinephrine, arteriosclerosis, or aortic coarctation showed that pulsatile components increased disproportionately and energy loss to pulsatility became more prominent (Porjé, 1967; O’Rourke, 1967; Nichols et al., 1986). In contrast, our study indicated preserved efficiency, which may reflect drug-specific effects (norepinephrine vs. phenylephrine) as well as species- or animal-model differences.

Since oscillatory power might reflect pulsatile aortic properties, whereas mean power is considered more closely related to vascular resistance (Saouti et al., 2010), the proportional rise in both components suggests balanced loading rather than selective worsening of pulsatile afterload. This suggests a strong effect of phenylephrine on steady vascular resistance on the one hand, while the concomitant rise in Ees and d*P*/d*t* suggests enhanced ventricular contractility on the other. The concomitant increase in Ees and d*P*/d*t* is also consistent with prior juvenile pig data showing higher steady load, preload, and contractility during phenylephrine exposure (Wodack et al., 2019).

No meaningful association emerged between Ea/Ees and OPF during phenylephrine administration, supporting the view that these metrics capture distinct, though partly related, aspects of ventriculoarterial interaction.

### 4.2 Nitroprusside

Nitroprusside markedly lowered arterial pressure, with a greater effect on Ea than on Ees, producing a clear reduction in Ea/Ees. This pattern is consistent with its predominantly afterload-reducing action described in heart failure and low-gradient aortic stenosis, where Ea falls while Ees is preserved or only modestly affected (Chantler et al., 2011; Lloyd et al., 2017).

Steady and pulsatile power declined in parallel, whereas OPF changed little and remained in the same range as during phenylephrine administration, again higher than values reported in healthy adults (Westerhof et al., 2019). In contrast to heart failure studies showing that nitroprusside lowers both resistance and pulsatile aortic impedance (Pepine et al., 1979), the present findings suggest a more balanced reduction of steady and pulsatile load in juvenile porcine hearts. A modest fall in contractility and stroke volume may reflect reduced preload from combined arterial and venous dilation, in keeping with earlier reports of slight negative inotropic effects of nitroprusside (Schlant et al., 1962; Roy et al., 1989). Such limited negative inotropy may help stabilize the fraction of energy lost to pulsatile components (and thus OPF) when stroke volume and pressure decline together, in line with reports that nitroprusside can modify relaxation and diastolic properties without major positive inotropic effects (Paulus et al., 1994).

Overall, nitroprusside primarily altered the steady component of afterload, while pulsatile efficiency showed only minor improvements. This again might emphasize that OPF and Ea/Ees are not interchangeable descriptors of the concept of ventriculoarterial interaction.

### 4.3 Esmolol

Esmolol produced a pronounced negative inotropic response, with parallel reductions in d*P*/d*t* and Ees and only a minor change in Ea, indicating a predominantly ventricular effect. Interestingly, in our study, heart rate remained constant. Accordingly, Ea/Ees increased, reflecting worsened ventriculoarterial coupling, consistent with previous porcine data (Monge García et al., 2020). This contrasts with septic shock studies in which esmolol induced a reduction in heart rate and, hence, improvements in arterial elastance and ventriculoarterial interaction after prior hemodynamic optimization (Morelli et al., 2013).

At the same time, total hydraulic energy delivery and stroke work declined, indicating lower global energy expenditure and myocardial oxygen demand, while OPF increased, suggesting relatively greater energy loss to pulsatility. Therefore, esmolol effectively reduced global energy expenditure and ventricular oxygen demand, but presumably impaired pulsatile efficiency.

The esmolol administration yielded the most informative result in terms of our initial research question. It was the one challenge in which both Ea/Ees and OPF changed substantially; the correlation between their changes was negative. Animals with the larger rise in Ea/Ees showed the smaller rise in OPF. Concordant changes at the group level therefore need not imply that the two indices track one another within individuals. With a minimal detectable |*r*| ≥ 0.73–0.79, we could unfortunately not determine whether there was a relevant relationship or just insufficient precision.

### 4.4 Dobutamine

Dobutamine elicited a marked positive inotropic response, with increased Ees and d*P*/d*t* consistent with β1-mediated enhancement of contractility (Freeman and Colston, 1990; Gayat et al., 2011; Razazi et al., 2022). Oscillatory and total power increased in parallel with stable OPF, indicating preserved pulsatile energy transmission despite higher cardiac work, in line with prior studies (Freeman and Colston, 1990; Nozawa et al., 1988).

Dobutamine-induced vasodilation limited the rise in Ea despite increased Ees, resulting in a lower Ea/Ees ratio and improved global ventriculoarterial coupling. This agrees with previous invasive and noninvasive studies showing that dobutamine increases contractility while leaving Ea unchanged or reduced, thereby shifting coupling toward a more favorable and mechanically efficient range (Freeman and Colston, 1990; Gayat et al., 2011; Ishizaka et al., 1988; Matsumoto et al., 2016). Dobutamine did not show a meaningful relationship between Ea/Ees and OPF.

### 4.5 Summary and clinical outlook

The concept of VAC integrates ventricular contractility and arterial load into a single measure of cardiovascular efficiency and performance (Monge García et al., 2020; Pinsky and Guarracino, 2020; Zhou et al., 2022).

There is now broad evidence that VAC by Ea/Ees ratio carries prognostic and therapeutic information beyond conventional parameters in heart failure, perioperative care and acute circulatory failure, and is also being explored as a therapeutic target in algorithm-guided resuscitation (Monge García et al., 2020; Andrei et al., 2022; Guinot et al., 2022; Aslanger et al., 2016; Chang et al., 1998; Antonini-Canterin et al., 2009; Demailly et al., 2023) and as an independent therapeutic goal in critical care (Guarracino et al., 2014, 2018, 2021).

On the other hand, experimental and clinical work has emphasized that pulsatile afterload (OPF) carries distinct physiological and pathophysiological information beyond conventional elastance-based coupling metrics. This is shown in previous work in which pulsatile work fraction increases with lower heart rate, higher flow, or reduced arterial distensibility, even when mean resistance is unchanged, underscoring its dependence on pulsatile rather than steady load (O’Rourke, 1967; O’Rourke and Taylor, 1967; N. Westerhof et al., 1972; Stergiopulos and Westerhof, 1999; Nico Westerhof et al., 2009; Chirinos, 2013; Coutinho et al., 2013; Chirinos and Sweitzer, 2017).

Ea/Ees and OPF describe different physiological domains of ventriculoarterial interaction. Ea/Ees is strongly influenced by mean arterial resistance and heart rate and much less by pulsatile properties of the arterial system. A large human study demonstrated that “Ea is simply a function of systemic vascular resistance and heart rate and is negligibly influenced by (and insensitive to) changes in pulsatile afterload in humans” (Chirinos et al., 2014). Ea/Ees describes a more globalized ventriculoarterial match over the entire heart cycle, including diastole (Chirinos and Sweitzer, 2017; Weber and Chirinos, 2018; Segers et al., 2002). In other words, Ea/Ees reflects the ratio between stroke work and potential energy, represents left ventricular energy efficiency, and is thus a pseudo-quantification of the relative amount of left ventricular energy losses.

By contrast, OPF appears to quantify energy lost to vascular pulsations and wave dynamics during systole, largely blind to diastolic runoff into the arterioles. Determinants of OPF in the systemic circulation are less well studied, but may include systemic vascular resistance, HR, and SV. In general, OPF may provide a more physiologically grounded description of pulsatile load and energy dissipation (Chirinos and Sweitzer, 2017; Andrei et al., 2022; Saeed et al., 2021; Dybos Tannvik et al., 2020).

In our current study, vasoactive and inotropic drugs widely used in intensive care modulated pulsatile systolic efficiency (OPF) and steady VAC (Ea/Ees) separately in a well-established animal model. Interestingly, our baseline Ea and Ees were higher than previously reported in adult porcine *in vivo* studies, where reported Ea ranged from 0.52 ± 0.14 mmHg mL^−1^ to 0.89 ± 0.16 mmHg mL^−1^ and Ees ranged from 0.30 ± 0.0.07 mmHg mL^−1^ to 0.42 ± 0.13 mmHg mL^−1^, with corresponding Ea/Ees ratios ranging from about 1.4 to 2.2 (Monge Garcia et al., 2018, 2019; Monge García et al., 2020).

In our present study, however, Ea/Ees ended up at 2.1 to 2.8. This range is considerably higher and indicates a more uncoupled state in juvenile pigs than in adult swine, dogs, or most human datasets. This possible increased level of uncoupling might be due to our population of juvenile pigs. In fact, available datasets were established in different animal cohorts or adult human populations (Miyagi et al., 2022; Little and Cheng, 1991; Little et al., 1989; Redfield et al., 2005) or in a more mature porcine population (double the age and size, different race) (Monge Garcia et al., 2018, 2019; Monge García et al., 2020).

Alternatively, this difference in Ea/Ees might be the result of methodological issues such as the effect of anesthetic drugs and/or positive-pressure ventilation on both arterial-aortic properties and ventricular performance, hence the Ea/Ees ratio. We used propofol/dexmedetomidine and ketamine in our protocol rather than isoflurane, as used in previous studies (Monge Garcia et al., 2018, 2019; Monge García et al., 2020), while older datasets were established by examining conscious animals (Little and Cheng, 1991; Little et al., 1989) or awake humans (Redfield et al., 2005).

Additionally, vendor-dependent measurement variations might have contributed to systematic errors (CD Leycom, Zoetermeer, Netherlands vs. Millar/AdInstruments, Dunedin, New Zealand).

However, all pharmacologic challenges yielded meaningful hemodynamic changes and, as the aim of the study was to track changes rather than absolute values, those differences between different studies might be considered less important.

Based on the findings of the current study, OPF cannot be considered a reliable surrogate for Ea/Ees in an experimental setting in juvenile pigs.

Rather, Ea/Ees and OPF should be viewed as complementary, non-interchangeable descriptors of ventriculoarterial interaction (Chirinos and Sweitzer, 2017; Tannvik et al., 2019; Weber and Chirinos, 2018; Segers et al., 2002). While Ea/Ees remains a validated integrative index, pressure–flow-derived power metrics may add physiological resolution in settings where pulsatile and steady components of afterload diverge, such as arterial stiffening or vasoactive drug therapy (Chirinos and Sweitzer, 2017; Andrei et al., 2022; Saeed et al., 2021; Dybos Tannvik et al., 2020). Combining both approaches may therefore improve bedside characterization of ventriculoarterial interaction and refine hemodynamic assessment (Monge García et al., 2020; Pinsky and Guarracino, 2020; Demailly et al., 2023).

### 4.6 Strengths and limitations

This study has several strengths. It presents a controlled, invasive large animal model on healthy animals with direct pressure–volume measurements and simultaneous invasive pressure and flow recordings, as well as indices of ventriculoarterial coupling obtained by imposing pharmacological challenges with predefined hemodynamic goals. It enabled time-resolved calculation of hydraulic power components, which has rarely been a focus in earlier animal studies and extends prior PV-based work that focused primarily on Ea/Ees and related elastance indices (Monge García et al., 2020; Seemann et al., 2023; Stonko et al., 2024).

However, some limitations should be acknowledged. All our measurements were performed in thoracotomized supine animals with an open thorax and without a respiratory hold. The aortic pressure catheter was placed in the descending aorta to avoid interference with the ascending aortic flow probe, requiring *post hoc* synchronization between pressure and flow and introducing potential bias compared with fully colocalized measurements (Seemann et al., 2023). Reassuringly, previous work has shown that minimally invasive ultrasound flow combined with more peripheral arterial pressure yields power integrals comparable to central measurements (Rimehaug et al., 2013).

Finally, this was a small study in juvenile pigs. Only correlations of |*r*| ≥ 0.73 would have been detectable, because the question concerns between-animal variation in the magnitude of the drug response and each animal therefore contributes one independent observation. Repeated recordings within an animal reduce the measurement error in its change score, for which we account, but cannot increase the number of animals. Our data are consequently inconclusive as to whether the two responses are coupled, and we draw no conclusion from that analysis.

The correlations between the two responses differed between interventions, including a change of sign, but with 10–12 animals per intervention, the study did not have sufficient power to establish whether such differences are real. Apparent differences between interventions in this dataset should accordingly be treated as hypothesis-generating.

As with all porcine studies, extrapolation to human physiology should be cautious given species- and age-related differences (Galbas et al., 2024; Miyagi et al., 2022; Lelovas et al., 2014).

## Data Availability

The raw data supporting the conclusions of this article will be made available by the authors, without undue reservation.
