# Supplementary material for: Ea/Ees and oscillatory power fraction capture complementary but distinct components of ventriculoarterial coupling: a porcine study
Source: Front Physiol. 2026 Aug 25;17:1904487. doi: 10.3389/fphys.2026.1904487 (PMC13550224; doi:10.3389/fphys.2026.1904487)
Supplement: Supplementary file 1 [file SupplementaryFile1.docx]

**Supplementary material**

Supplementary Document 1: Experimental Protocol. VC, volume controlled ventilation; TV, tidal volume; pCO2, partial pressure of CO2; RR, respiratory rate; aBG, arterial blood gas; IV, intravenously; IM, intramuscularly; Fr, French; IAP Mx, intraarterial pressure measurement; CVL, central venous line; IJV, internal jugular vein; 3wt, three-way-tap; FBC, Fogerty balloon catheter; UL, ultrasound; LVOT, left ventricular outflow tract; AV, aortic valve; PV loop, pressure-volume-loop; BL Mx, baseline measurement; Effect Mx, effect measurement; OPF, oscillatory power fraction; Ea, arterial elastance; Ees, endsystolic elastance; OPF_N_BL_(drug)_1-3, measurement 1 to 3 of OPF in normal heart condition at BL with given drug (phenylephrine/nitroprusside/esmolol/dobutamine); EES_N_BL_(drug)_1-3, measurement 1 to 3 of Ees in normal heart condition at BL before given drug (phenylephrine/nitroprusside/esmolol/dobutamine); OPF_N_EF_(drug)_1-3, measurement 1 to 3 of OPF in normal heart condition at effect with given drug (phenylephrine/nitroprusside/esmolol/dobutamine); Ees_N_EF_(drug)_1-3, measurement 1 to 3 of Ees in normal heart condition at effect with given drug (phenylephrine/nitroprusside/esmolol/dobutamine); IVC, inferior vena cava; MAP, mean arterial pressure;

Supplementary Table 1: Correlation between OPF and Ea/Ees computed on values averaged within each animal, separately at baseline and at the drug-effect state. These are cross-sectional correlations between animals at a single state and do not describe whether the two indices change together (Table 5). With 10–12 animals only correlations of |r| ≥ 0.73–0.79 were detectable at 80% power. Together with the change-score coefficients in Table 5 these form the twelve correlations across which the Benjamini-Hochberg adjustment was applied. n, number of animals; OPF, oscillatory power fraction; Ea/Ees, ventriculo-arterial coupling by ratio of elastances; r, Pearson's correlation coefficient; CI, confidence interval; p (BH), significance level after Benjamini-Hochberg adjustment.

| **Intervention** | **State** | **n** | **r (95% CI)** | **p** | **p (BH)** |
| --- | --- | --- | --- | --- | --- |
| Phenylephrine | Baseline | 12 | -0.21 (-0.70 to +0.41) | 0.512 | 0.592 |
| Nitroprusside | Baseline | 12 | +0.21 (-0.41 to +0.70) | 0.513 | 0.592 |
| Esmolol | Baseline | 10 | -0.33 (-0.79 to 0.38) | 0.352 | 0.591 |
| Dobutamine | Baseline | 10 | −0.33 (−0.79 to +0.38) | 0.358 | 0.591 |
| Phenylephrine | Drug effect | 12 | −0.20 (−0.69 to +0.43) | 0.543 | 0.592 |
| Nitroprusside | Drug effect | 11 | +0.14 (−0.50 to +0.68) | 0.679 | 0.679 |
| Esmolol | Drug effect | 10 | −0.69 (−0.92 to −0.11) | 0.026 | 0.219 |
| Dobutamine | Drug effect | 10 | −0.30 (−0.78 to +0.40) | 0.394 | 0.591 |

Supplementary Table 2: Smallest correlation each analysis could have identified with 80% power at a two-sided significance level of 0.05, computed from the effective sample size via Fisher's *z* transformation. For the animal-averaged and change-score analyses the effective sample size is the number of animals, because both concern variation between animals. For the repeated-measures correlation it is the residual degrees of freedom, but that analysis addresses a different question (see 2.4.3). Post-hoc observed power is not reported, being a deterministic function of the observed significance level.

| **Analysis** | **Intervention** | **Effective n** | **Smallest detectable \|r\|** |
| --- | --- | --- | --- |
| Animal-averaged correlation | PE, NP | 12 | 0.73 |
| Animal-averaged correlation | ES, DO | 10 | 0.79 |
| Change-score correlation | PE | 12 | 0.73 |
| Change-score correlation | NP | 11 | 0.76 |
| Change-score correlation | ES, DO | 10 | 0.79 |
| Repeated-measures correlation | PE | 58 | 0.36 |
| Repeated-measures correlation | NP | 54 | 0.37 |
| Repeated-measures correlation | ES | 49 | 0.39 |
| Repeated-measures correlation | DO | 51 | 0.38 |

Supplementary Table 3: Partial correlation between OPF and Ea/Ees after adjustment for animal and for measurement state, using all paired observations (Bakdash and Marusich, 2017). Because each animal was measured in only two states, this quantity conditions out the average effect of the intervention and therefore does not describe whether OPF tracks drug-induced changes in Ea/Ees. It is reported for completeness rather than as evidence bearing on the study question. Abbreviations as in Supplementary Table 1.

| **Intervention** | **Observations** | **Animals** | **r (95% CI)** | **p** |
| --- | --- | --- | --- | --- |
| Phenylefrine | 69 | 12 | −0.30 (−0.52 to −0.05) | 0.023 |
| Nitroprusside | 65 | 12 | +0.44 (+0.19 to +0.63) | 0.001 |
| Esmolol | 58 | 10 | −0.52 (−0.70 to −0.28) | <0.001 |
| Dobutamine | 60 | 10 | −0.31 (−0.54 to −0.04) | 0.026 |
